# Anti-Infectivity against Herpes Simplex Virus and Selected Microbes and Anti-Inflammatory Activities of Compounds Isolated from *Eucalyptus globulus* Labill.

**DOI:** 10.3390/v10070360

**Published:** 2018-07-06

**Authors:** Viliam Brezáni, Veronika Leláková, Sherif T. S. Hassan, Kateřina Berchová-Bímová, Pavel Nový, Pavel Klouček, Petr Maršík, Stefano Dall’Acqua, Jan Hošek, Karel Šmejkal

**Affiliations:** 1 Department of Natural Drugs, Faculty of Pharmacy, University of Veterinary and Pharmaceutical Sciences Brno, Palackého třída 1946/1, 612 42 Brno, Czech Republic; brezani.viliam@gmail.com (V.B.); sherif.hassan@seznam.cz (S.T.S.H.); 2 Department of Molecular Biology and Pharmaceutical Biotechnology, Faculty of Pharmacy, University of Veterinary and Pharmaceutical Sciences Brno, Palackého třída 1946/1, 612 42 Brno, Czech Republic; veronika.lelakova@gmail.com (V.L.); hosekj@vfu.cz (J.H.); 3 Department of Applied Ecology, Faculty of Environmental Sciences, Czech University of Life Sciences Prague, Kamýcká 129, 165 21 Praha 6-Suchdol, Czech Republic; berchova@knc.czu.cz; 4 Department of Quality of Agricultural Products, Faculty of Agrobiology, Food and Natural Resources, Czech University of Life Sciences Prague, Kamýcká 129, 165 21 Praha 6-Suchdol, Czech Republic; novy@af.czu.cz (P.N.); kloucek@af.czu.cz (P.K.); marsik@af.czu.cz (P.M.); 5 Department of Pharmaceutical and Pharmacological Sciences, University of Padua, Via F. Marzolo 5, 351 31 Padua, Italy; stefano.dallacqua@unipd.it

**Keywords:** *Eucalyptus*, antiherpetic, HSV-1, HSV-2, antibacterial, anti-inflammatory, NF-κB/AP-1, IL-1β, TNF-α, ROS

## Abstract

Herpes simplex virus (HSV) causes numerous mild-to-serious human diseases, including mucocutaneous herpes infections and life-threatening herpes encephalitis. Moreover, herpes viral lesions can be complicated by inflammation and secondary bacterial infections. The development of resistance to antiviral drugs along with the undesirable side effects of these drugs are relevant argue for the development of new anti-HSV drugs with diverse mechanisms of action. *Eucalyptus* extracts have been used for decades to combat various infectious diseases. We isolated and studied 12 pure compounds and one mixture of two constitutional isomers from the leaves and twigs of *E. globulus*. The structures were identified by spectroscopic methods (NMR, HR-MS, IR) and all of them were tested for antiherpetic activity against the replication of antigen types HSV-1 and HSV-2. Tereticornate A (**12**) (IC_50_: 0.96 μg/mL; selectivity index CC_50_/IC_50_: 218.8) showed the strongest activity in the anti-HSV-1 assay, even greater than acyclovir (IC_50_: 1.92 μg/mL; selectivity index CC_50_/IC_50_: 109.4), a standard antiviral drug. Cypellocarpin C (**5**) (EC_50_: 0.73 μg/mL; selectivity index CC_50_/EC_50_: 287.7) showed the most potent anti-HSV-2 activity, also more intensive than acyclovir (EC_50_: 1.75 μg/mL; selectivity index CC_50_/EC_50_: 120.0). The antimicrobial activity of the isolated compounds was also evaluated against the bacteria *Staphylococcus aureus*, *Bacillus cereus*, *Escherichia coli*, and *Pseudomonas aeruginosa* and the yeast *Candida albicans*. The anti-inflammatory potential was examined using LPS-stimulated THP-1-XBlue™-MD2-CD14 and THP-1 macrophages and focusing on the influences of the NF-κB/AP-1 activity and the secretion of pro-inflammatory cytokines IL-1β and TNF-α.

## 1. Introduction

*Eucalyptus* is a large genus of the Myrtaceae family; more than 900 species and subspecies have been described. This evergreen tree occurs naturally in Australia [1] and since the 19th century *Eucalyptus* trees have been harvested in southeastern Tasmania and shipped throughout the world for use as wharf piles. The timber has also been used for mine supports, railway sleepers, and street-paving blocks [2]. *Eucalyptus* trees have been introduced worldwide to regions where the climate suits them [3]. The *Eucalyptus* is a fast-growing tree suitable for making paper [4]. Although it is grown in many countries around the world, Australia is the only place where *Eucalyptus* trees dominate the woodlands. The Aborigines developed a sophisticated empirical understanding of the healing potential of *Eucalyptus*. Traditionally, they used its leaves to treat wounds, colds, influenza, toothaches, snakebites, fevers, diarrhea, and other complaints [1,5]. Kino, the astringent exudate produced following a pathological or mechanical injury to the wood, has been used as a powder or paste to treat open sores and as an aqueous solution for external and internal diseases [5]. *Eucalyptus* soon spread to Chinese, Indian, and European phytotherapy. It is used in many countries to treat a variety of medical conditions [2,6]. A hot-water extract of the dried leaves of *E. globulus* is used traditionally as a remedy to treat symptoms of respiratory infections, such as cold, flu, and sinus congestion [7,8,9]. “Eucalyptus leaf extract” has been approved as a natural food additive and it is included among the antioxidants in the “List of Existing Food Additives in Japan” [9]. *Eucalyptus* extracts have shown various biological effects, such as antibacterial, antifungal, antioxidant, or anti-hyperglycemic activities [4,9]. Extracts of the leaves of *E. sideroxylon* and *E. camaldulensis* have exerted antiviral activity against herpes simplex virus (HSV) [10,11].

HSV is a large, enveloped, double-stranded DNA virus of the Herpesviridae family [12]. This important human pathogen is differentiated into two distinct antigen types: HSV-1 and HSV-2 [13]. Both types are responsible for a variety of mild to serious human diseases such as mucocutaneous herpes infections, herpes keratitis, herpetic encephalitis, and neonatal herpes [11,14]. Common areas of HSV-1 infection are the face, lips, mouth cavity, and the skin of the loin and the area above the loin, whereas HSV-2 affects mainly the genital area and the skin of the area below the loin [15]. Life-threatening herpes infections are those that affect neonates, elderly people, immune-compromised patients, transplant patients under immune suppression, and patients with acquired immune deficiency syndrome [11]. The principal mode by which herpes infections spread is direct contact with infected secretions [12]. The cells of the mucocutaneous surface are the site of entry for HSV infection [13]. Following an acute infection, retrograde transport via sensory nerve endings carries particles of HSV to ganglia, where the virus establishes latency until it is reactivated by a stimulus. The recurrence of the virus infection occurs by centrifugal spreading of the HSV in axons from the ganglia followed by replication at the mucosal surface [13,14]. Nucleoside analogues, such as acyclovir and its derivatives—foscarnet and cidofovir—are used to treat HSV infections. The mechanism of their activity is based on inactivation of the viral DNA polymerase [13,14,15]. The development of resistance to antiviral agents, along with the undesirable side effects of nucleoside analogs argue for the development of new anti-HSV drugs with diverse mechanisms of action [11,13].

The genus *Eucalyptus* is a rich source of biologically active compounds. Two main groups of molecules can be distinguished: volatile and non-volatile compounds [16]. The volatile compounds present in *Eucalyptus* essential oils possess antibacterial and antiviral activities [9,14,15]. Non-volatile compounds comprise a miscellaneous group of secondary metabolites. Some of them have shown various biological activities, including anti-inflammatory, antioxidant, antibacterial, and cytotoxic activity [16]. None of them have shown significant antiviral activity against HSV. In this work, we tested compounds isolated from an ethanolic extract obtained from the leaves and twigs of *E. globulus* to identify the antiviral activity against both *H. simplex* antigen types, HSV-1 and HSV-2. We also measured their antimicrobial potentials against the bacteria *S. aureus*, *B. cereus*, *E. coli*, and *P. aeruginosa* and the yeast *C. albicans* and determined their anti-inflammatory and antioxidant potentials.

## 2. Materials and Methods 

### 2.1. Phytochemistry

#### 2.1.1. Plant Material

Leaves and twigs of *E. globulus* were acquired from the Centrum of Medicinal Plants of the Medical Faculty of Masaryk University in Brno. The plant was identified by Ing. Pavel Musil (head of Centrum of Medicinal Plants). The material was harvested and air-dried during the summer of 2010. Voucher specimen EG-2010 was deposited at the Herbarium of the Department of Natural Drugs, Faculty of Pharmacy, UVPS Brno.

#### 2.1.2. General Experimental Procedure

NMR spectroscopy (^1^H, HSQC, HMBC, COSY, NOESY) was carried out on a Bruker Avance III 400 spectrometer (Bruker, Billerica, MA, USA) with TMS as the internal standard. Norell^®^ Standard Series™ 5 mm NMR tubes (Norell, Morganton, NC, USA) were employed. CDCl_3_-*d* (Sigma-Aldrich, Steinheim am Albuch, Germany), MeOD-*d*4 (Sigma-Aldrich) and DMSO_4_-*d*6 (Sigma-Aldrich) were used as solvents. HRMS spectra in the positive mode were measured using an LC/MS instrument consisting of a Q-TOF mass spectrometer with ultra-high resolution and mass accuracy Impact II (Bruker Daltonics GmbH, Bremen, Germany) combined with a UHPLC UltiMate 3000 separation system (Thermo Fischer Scientific, Waltham, MA, USA). Infrared spectra were obtained on an FT-IR Nicolet Impact 410 spectrometer with Omnic software (Nicolet, Waltham, MA, USA), using the ATR technique in the solid state. Preparative HPLC was performed using a DIONEX UltiMate 3000 instrument (Thermo Scientific, Waltham, MA, USA) with an UltiMate 3000 Collector, or YL 9100 HPLC System (Young Lin, Anyang-si, Korea) with a FOXY R2 Fraction Collector (Teledyne Isco, Lincoln, NE, USA), employing an Ascentis RP-Amide, 25 cm × 10 mm, particle size 5 μm (Sigma-Aldrich) semipreparative HPLC column. Analytical HPLC measurements were obtained on an Agilent 1100 with a diode array detector, using an analytical Ascentis Express RP-Amide (15 cm × 2.1 mm, particle size 2.7 μm (Sigma-Aldrich)) column. Column chromatography was performed on silica gel with a particle size of 40–63 μm (Merck, Kenilworth, NJ, USA). Silica gel 60 F_254_ 20 × 20 cm, 200 μm (Merck) TLC plates were used for analytical purposes. Deionized water was obtained using an Aqua MAX BASIC 360 series and Aqua MAX ULTRA 370 series water purification system. Acetic acid 98% p.a. (Ing. Petr Švec-Penta, Chrudim, Czech Republic), acetone p.a. (Ing. Petr Švec-Penta), benzene p.a. (Ing. Petr Švec-Penta), ethyl-acetate p.a. (Ing. Petr Švec-Penta), chloroform p.a. (Ing. Petr Švec-Penta), and methanol p.a. (Ing. Petr Švec-Penta) were used for column chromatography and TLC. Acetonitrile HPLC ultra gradient grade (J.T. Baker, Avantor, Gliwice, Poland), methanol HPLC gradient grade (J.T. Baker), deionized water and formic acid 98% p.a. (Ing. Petr Švec-Penta) were used for preparative chromatography.

#### 2.1.3. Extraction and Isolation

Leaves and twigs of *E. globulus* (2500 g) were macerated in 96% ethanol. After the solvent was removed, the crude extract (567 g) was dissolved in aqueous methanol and extracted with *n*-hexane was used to eliminate unwanted lipophilic compounds. Subsequent extraction of the aqueous methanol with chloroform, followed by drying, yielded 83 g of solid material that was thought to contain the largest amount of the compounds with balance of lipophilic and hydrophilic features, e.g., phloroglucinol-terpene adducts or *C*-methylated flavonoids. Column chromatography with chloroform/methanol/benzene (8/1/1, *v*/*v*/*v*) was used for a rough separation of the chloroform extract. Fractions containing desirable compounds were then submitted to a second stage of separation using column chromatography with the mobile phase depending on the results of the TLC analysis. Finally, those fractions that showed the presence of a major compound the fractions were purified using preparative HPLC.

#### 2.1.4. Isolated Compounds

Litseagermacrane (**1**): colorless gum; UV (CH_3_OH) λ_max_ 225, 245 nm; IR (ATR) υ_max_ 3373(br), 2951, 2870, 1709, 1667, 1442, 1362, 1282, 1163, 1066, 1033, 978, 920 cm^−1^; ^1^H and ^13^C NMR, HMBC spectral data see Table S1, COSY and NOESY correlations see Figure S1; HRMS *m*/*z* 237.1844 [M + H]^+^ (calcd for C_15_H_25_O_2_^+^, 237.1849), 259.1663 [M + Na]^+^ (calcd for C_15_H_24_NaO_2_^+^, 259.1669).

Grandinol (**2**): yellowish powder; UV (CH_3_OH) λ_max_ 276, 345 nm; IR (ATR) υ_max_ 3160(br), 2955, 2871, 1622, 1435, 1382, 1308, 1188, 949, 872, 830, 772 cm^−1^; ^1^H and ^13^C NMR, HMBC spectral data see Table S2, COSY correlations see Figure S2; HRMS *m*/*z* 253.1072 [M + H]^+^ (calcd for C_13_H_17_O_5_^+^, 253.1071), *m*/*z* 275.0891 [M + Na]^+^ (calcd for C_13_H_16_NaO_5_^+^, 275.0890).

Pulverulentone B (**3**): pale yellow powder, UV (CH_3_OH) λ_max_ 240, 295 nm; IR (ATR) υ_max_ 3204(br), 2933, 2873, 1607, 1421, 1228, 1206, 1119, 1057, 803 cm^−1^; ^1^H and ^13^C NMR, HMBC spectral data see Table S3, COSY and NOESY correlations see Figure S3; HRMS *m*/*z* 239.1282 [M + H]^+^ (calcd for C_13_H_19_O_4_^+^, 239.1278).

Eucalyptal A (**4**): pale yellow powder, UV (CH_3_OH) λ_max_ 276 nm; IR (ATR) υ_max_ 3405, 2928, 2666, 1625, 1436, 1382, 1303, 1267, 1191, 1155, 1068, 1032, 995, 907, 869, 813 cm^−1^; ^1^H and ^13^C NMR, HMBC spectral data see Table S4, COSY and NOESY correlations see Figure S4; HRMS *m*/*z* 469.2584 [M + H]^+^ (calcd for C_28_H_37_O_6_^+^, 469.2585), *m*/*z* 491.2403 [M + Na]^+^ (calcd for C_28_H_36_NaO_6_^+^, 491.2404).

Cypellocarpin C (**5**): pale pink powder; UV (CH_3_OH) λ_max_ 238, 255, 285, 315 nm; IR (ATR) υ_max_ 3313(br), 2972, 2918, 2882, 1705, 1659, 1619, 1581, 1414, 1378, 1343, 1246, 1062, 1018, 842 cm^−1^; ^1^H and ^13^C NMR, HMBC spectral data see Table S5, COSY correlations see Figure S5; HRMS *m*/*z* 521.2026 [M + H]^+^ (calcd for C_26_H_33_O_11_^+^, 521.2017), *m*/*z* 543.1848 [M + Na]^+^ (calcd for C_26_H_32_NaO_11_^+^, 543.1837).

Sideroxylin (**6**): yellow powder; UV (CH_3_OH) λ_max_ 208, 278, 328 nm; IR (ATR) υ_max_ 3582, 3512, 3070, 2925, 1717, 1643, 1602, 1570, 1541, 1447, 1329, 1251, 1214, 1176, 1106, 1034, 977, 956, 831 cm^−1^; ^1^H and ^13^C NMR, HMBC spectral data see Table S6, COSY and NOESY correlations see Figure S6; HRMS *m*/*z* 313.1070 [M + H]^+^ (calcd for C_18_H_17_O_5_^+^, 313.1071), *m*/*z* 335.0888 [M + Na]^+^ (calcd for C_18_H_16_NaO_5_^+^, 335.0890).

8-Demethylsideroxylin (**7**): yellow powder; UV (CH_3_OH) λ_max_ 214, 274, 332 nm; IR (ATR) υ_max_ 3731, 3133, 2924(br), 1652, 1600, 1558, 1490, 1444, 1348, 1297, 1247, 1173, 1137, 1030, 825 cm^−1^; ^1^H and ^13^C NMR, HMBC spectral data see Table S7, COSY and NOESY correlations see Figure S7; HRMS *m*/*z* 299.0919 [M + H]^+^ (calcd for C_17_H_15_O_5_^+^, 299.0914), *m*/*z* 321.0738 [M + Na]^+^ (calcd for C_17_H_14_NaO_5_^+^, 321.0733).

Eucalyptin (**8**): yellow powder; UV (CH_3_OH) λ_max_ 200, 284, 326 nm; IR (ATR) υ_max_ 3353(br), 3075, 2918, 2836, 1650, 1569, 1508, 1423, 1361, 1333, 1252, 1212, 1181, 1112, 1026, 981, 956, 825 cm^−1^; ^1^H and ^13^C NMR, HMBC spectral data see Table S8, COSY and NOESY correlations see Figure S8; HRMS *m*/*z* 327.1223 [M + H]^+^ (calcd for C_19_H_19_O_5_^+^, 327.1227), 349.1040 [M + Na]^+^ (calcd for C_19_H_18_NaO_5_^+^, 349.1046).

8-Demethyleucalyptin (**9**): pale yellow crystals; UV (CH_3_OH) λ_max_ 215, 276, 331 nm; ; IR (ATR) υ_max_ 2920(br), 1663, 1595, 1508, 1427, 1350, 1308, 1261, 1133, 1027, 906, 828, 795 cm^−1^; ^1^H and ^13^C NMR, HMBC spectral data see Table S9, COSY and NOESY correlations see Figure S9; HRMS *m*/*z* 313.1077 [M + H]^+^ (calcd for C_18_H_17_O_5_^+^, 313.1071), 335.0896 [M + Na]^+^ (calcd for C_18_H_16_NaO_5_^+^, 335.0890).

Sesamin (**10**): white crystals; UV (CH_3_OH) λ_max_ 238, 286 nm; IR (ATR) υ_max_ 3316(br), 2899(br), 2848, 1712(br), 1440, 1364, 1247, 1192, 1092, 1055, 1032, 968, 924, 855, 801, 781, 750 cm^−1^; ^1^H and ^13^C NMR, HMBC spectral data see Table S10, COSY and NOESY correlations see Figure S10; HRMS *m*/*z* 377.0997 [M + Na]^+^ (calcd for C_20_H_18_NaO_6_^+^, 377.0996), *m*/*z* 393.0736 [M + K]^+^ (calcd for C_20_H_18_KO_6_^+^, 393.0735).

Ursolic acid (**11**): white powder; IR (ATR) υ_max_ 2925, 1689, 1456, 1387, 1042, 997 cm^−1^; ^1^H and ^13^C NMR, HMBC spectral data see Table S11, COSY and NOESY correlations see Figure S11; HRMS *m*/*z* 457.3683 [M + H]^+^ (calcd for C_30_H_49_O_3_^+^, 457.3676), *m*/*z* 479.3503 [M + Na]^+^ (calcd for C_30_H_48_NaO_3_^+^, 479.3496).

Tereticornate A (**12**): white powder; UV (CH_3_OH) λ_max_ 242, 297, 324 nm; IR (ATR) υ_max_ 3170 (br), 2924, 2853, 1735, 1697, 1627, 1590, 1513, 1451, 1386, 1360, 1263, 1217, 1162, 1142, 990, 903, 862 cm^−1^; ^1^H and ^13^C NMR, HMBC spectral data see Table S11, COSY and NOESY correlations see Figure S11; HRMS *m*/*z* 631.4001 [M + H]^+^ (calcd for C_40_H_55_O_6_^+^, 631.3993), 653.3820 [M + Na]^+^ (calcd for C_40_H_54_NaO_6_^+^, 653.3813).

Ursolic acid lactone (**13a**) and loxanic acid (**13b**): white powder; IR (ATR) υ_max_ 3266(br), 2929, 2856, 1760, 1447, 1386, 1359, 1219, 1138, 989, 902, 865 cm^−1^; ^1^H and ^13^C NMR, HMBC spectral data see Tables S13 and S14, COSY and NOESY correlations see Figures S13 and S14; HRMS *m*/*z* 455.3516 [M + H]^+^ (calcd for C_30_H_47_O_3_^+^_,_ 455.3520), *m*/*z* 477.3332 [M + Na]^+^ (calcd for C_30_H_46_NaO_3_^+^, 477.3339).

### 2.2. Antiherpetic Activity

All isolated compounds that were reported in this study, have been investigated for their inhibitory properties against HSV-1 and HSV-2 replication. Only those compounds with antiherpetic properties are reported in the methodology and results.

#### 2.2.1. Viral Strains, Cultures, Cell Lines and Reagents

Vero cells (ATCC: CCL 81™; UK), obtained from Motol University Hospital (Prague, Czech Republic), were grown in Eagle’s minimum essential medium (MEM; Cultilab, Campinas, UK) supplemented with 10% fetal bovine serum (FBS; Gibco, Carlsbad, CA, USA), penicillin G (100 U/mL), streptomycin (100 μg/mL), and amphotericin B (25 μg/mL) (Sigma-Aldrich, Germany) and maintained at 37 °C in a 5% CO_2_ atmosphere. The HSV-1 strain KOS, obtained from Motol University Hospital (Prague, Czech Republic), was propagated in Vero cells. Viral stocks were stored at −80 °C and titrated based on the plaque forming units (PFU) count by plaque assay, as described previously [17]. Ten clinical isolates of HSV-2 (isolated from patients with HSV-2 infections) were obtained from Motol University Hospital (Prague, Czech Republic). All clinical isolates were typed by a quantitative real-time reverse transcription PCR using primer pairs H_2_M_40_ 5′-GTACAGACCTTCGGAGG-3′ and H_2_P_4_ 5′-CGCTTCATCATGGGC-3′ for the identification. They were subsequently propagated in Vero cells. Viral stocks were stored at −80 °C. A cytopathic end-point assay was used to determine the titers expressed as 50% tissue culture infective dose (TCID_50_/mL) as has previously been described [18].

#### 2.2.2. Determination of Cytotoxicity

The cytotoxicity of compounds was evaluated by using the neutral red dye-uptake method as previously described [19,20]. Briefly, the test compounds **1**–**12**, along with **13** (**a** + **b**) and acyclovir (Sigma-Aldrich, Germany), used as a positive control were diluted in 0.1% dimethyl sulfoxide (DMSO). Stock solutions were prepared at a concentration of 420 μg/mL using deionized water and then sterilized. Vero cell monolayers cultivated in 96-well microtiter plates with two-fold serial dilutions of the test compounds or the positive control were incubated for 84 h at 37 °C in 5% CO_2_ atmosphere. After incubation, the morphological alterations of the treated cells were determined using an inverted optical microscope (Leitz, Berlin, Germany) and the maximum non-toxic concentrations (MNTC) were determined. The CC_50_ values (concentrations of each drug required to reduce the cell viability by 50%) of compounds **1**–**12**, along with **13** (**a** + **b**) and acyclovir were calculated as compared with the untreated control cells. The cytotoxicity of compounds **1**–**2**, **5**–**6**, and **12**, active in further anti-HSV tests, was evaluated in duplicate in at least three independent experiments to obtain statistically relevant data for final calculations.

#### 2.2.3. Anti-HSV-1 Assay

A plaque reduction assay with acyclovir as the positive control was used to evaluate the anti-HSV-1 activity [18,21]. Cell monolayers were infected with 100 PFU of HSV-1 in MEM containing 1.5% carboxymethyl cellulose (CMC, Sigma-Aldrich, Germany) in the presence or absence of active compounds **2**, **6**, and **12** at different concentrations. The cells were incubated for 72 h at 37 °C, then fixed and stained using naphthol blue black (Sigma-Aldrich) and finally the plaques were counted. The concentrations of compounds **2**, **6**, and **12** required to reduce the number of plaques by 50% (IC_50_) were calculated as compared with untreated control cells. A selectivity index was calculated as the ratio CC_50_/IC_50_.

#### 2.2.4. Anti-HSV-2 Assay

Anti-HSV-2 activities were determined by titer reduction assay as previously described [19]. Vero cell monolayers were treated with active compounds **1** and **5** and acyclovir as a positive control at concentrations for which no changes were observed in the cell morphology and the 80% level of cell viability was determined. 100 TCID_50_/mL of HSV-2 acyclovir-sensitive suspensions was added to the treated and untreated cell cultures and they were incubated at 37 °C for 48 h in a 5% CO_2_ atmosphere. After incubation, the virus titers in the treated and untreated cells were determined. The anti-HSV activity was evaluated as the percentage inhibition (PI) using antilogarithmic TCID_50_ values as follows: PI = [1 − (antilogarithmic test value/antilogarithmic control value)] × 100. The dose-response curve was determined from the MNTC and the 50% effective concentration (EC_50_) was determined as the concentration required for 50% protection against virus-induced cytopathic effects. Finally, a selectivity index was calculated as the ratio CC_50_/EC_50_.

#### 2.2.5. Statistical Analysis

PRISM software version 5.0 (GraphPad Software, Inc., San Diego, CA, USA) was used for statistical analysis and calculations. Experiments were carried out in duplicate in at least three independent experiments and the ± standard deviations (SD) from the mean values were calculated. Nonlinear regressions of the concentration-response curves were used to determine the CC_50_ and IC_50_ values from the HSV-1 assay. Anova/Dunnett/SNK tests were used to evaluate the significance of the test compounds and the positive control and the selectivity indexes CC_50_/IC_50_ of the test compounds were determined. The one-way ANOVA test was used for the statistical analysis and calculation of the CC_50_ and EC_50_ parameters of the HSV-2 assay. Finally, the selectivity index CC_50_/EC_50_ was determined.

### 2.3. Antibacterial and Antifungal Activity

#### Microorganisms and Cultivation Media

Microorganisms were selected to include Gram-positive and Gram-negative bacteria and a yeast. Bacterial strains *Staphylococcus aureus* ATCC 29313, *Bacillus cereus* ATCC 11778, *Escherichia coli* ATCC 25922 and *Pseudomonas aeruginosa* ATCC 27853, along with the yeast *Candida albicans* ATCC 10321 were purchased from Oxoid (Basingstoke, UK). The bacteria were grown and tested in Mueller–Hinton broth and the yeast in Sabouraud liquid medium from Oxoid (Basingstoke, UK). The antimicrobial activity was tested by the broth microdilution method, according to CLSI guidelines (CLSI2012; NCCLS 2002) with some modifications. The test compounds were dissolved in DMSO and diluted in the cultivation medium so that the maximum DMSO concentration was 1%. Because only limited amounts of the test compounds were available, their starting concentrations varied from 128 to 32 μg/mL (Table 3). Microplates were inoculated with a suspension of the bacteria and yeast standardized from the overnight culture to achieve final concentration of 5 × 10^5^ and 2 × 10^3^ CFU for bacteria and yeast, respectively. The results were evaluated after incubations of 24 h and 48 h at 35 ± 2 °C and 25 ± 2 °C for bacteria and yeast, respectively. The lowest concentration that was able to inhibit any visible growth was designated as the minimum inhibitory concentration (MIC). The compounds were tested in triplicate in three independent assays.

### 2.4. Anti-Inflammatory Activity

#### 2.4.1. Cell Culture

The THP-1 human monocytic leukemia cell line was purchased from the European Collection of Cell Cultures (Salisbury, UK) and the THP-1-XBlue™-MD2-CD14 cell line from Invivogen (San Diego, CA, USA). The cells were cultured in RPMI 1640 medium containing 2 mM stabilized l-glutamine (Biosera, Nuaille, France), supplemented with 10% (*v*/*v*) FBS (HyClone, Logan, UT, USA) and antibiotics (100 U/mL penicillin, 100 mg/mL streptomycin) (Sigma-Aldrich). The cultures were kept in an incubator at 37 °C in a water-saturated atmosphere of air containing 5% CO_2_. The cells were passaged approximately twice a week. Stabilized cells (5th–15th passage) were washed with PBS, put in serum-free medium and split into 96-well microtiter plates. These cells were then used for the experiments.

#### 2.4.2. Cell Viability Testing

THP-1 cells, floating monocytes at a concentration of 5 × 10^4^ cells/well were incubated at 37 °C in serum-free RPMI 1640 medium for 2 h. The cells were then treated with compounds **1**–**12** and **13** (**a** + **b**) dissolved in DMSO at increasing concentrations and cell viability was measured after 24 h. The maximum concentration of DMSO in the assay never exceeded 0.1%. Viability was measured by using the cell proliferation reagent WST-1 (Roche, Basel, Switzerland) according to the manufacturer’s manual. The amount of formazan corresponding to the number of metabolically active cells in the culture was calculated as a percentage of the control cells, which were treated only with the vehicle and were assigned as 100%. The IC_50_ values were calculated from resultant viability curves by four-parameter logistic (4PL) analysis.

#### 2.4.3. Detection of the Activation of NF-κB/AP-1

The activity of transcriptional factors NF-κB/AP-1 was evaluated on the THP-1-XBlue™-MD2-CD14 cell line expressing an NF-κB/AP-1-inducible secreted embryonic alkaline phosphatase (SEAP) reporter gene. Cells were placed into serum-free medium (500,000 cells/mL) and split into 96-well plates. After 2 h of incubation they were treated with test compounds **1**–**3**, **5**–**12**, and **13** (**a** + **b**) at a concentration of 5 μM, prednisone 5 μM, or only the vehicle. One hour later, lipopolysaccharide (LPS) from *E. coli* 0111:B4 (Sigma-Aldrich) dissolved in PBS was added to the treated cells at a final concentration of 1 μg/mL. LPS stimulation of the Toll-like 4 receptors in this cell line induced signaling cascades leading to the activation of NF-κB/AP-1 and the subsequent production of SEAP was measured after 24 h. The plates were centrifuged, 20 μL of the cultivation medium was mixed with 175 μL of Quanti-Blue™ reagent (Invivogen) and incubated according to the manufacturer’s instructions at 37 °C for 30–40 min. The activity of NF-κB/AP-1 was determined spectrophotometrically in a FLUOstar Omega microplate reader (BMG Labtech, Ortenberg, Germany) at λ 655 nm and compared with that of the vehicle.

#### 2.4.4. Differentiation into Macrophages and Evaluation of Cytokine Secretion

To differentiate THP-1 monocytes into macrophages, the cells were stimulated with phorbol myristate acetate (PMA) at a final concentration of 50 ng/mL. A suspension of cells at a concentration of 500,000 cells/mL in complete RPMI 1640 medium was split into the wells of a 96-well plate. The culture medium of adherent macrophages was replaced with fresh medium after 24 h. After another 24 h, the cells were washed with PBS, put into serum-free medium for 2 h and then used for subsequent experiments. Differentiated THP-1 macrophages were pre-treated for 1 h with 5 μM solutions of the test compounds **8** and **12**, a 1 μM solution of prednisone dissolved in DMSO or only the vehicle (0.1% (*v*/*v*) DMSO solution). The inflammatory-like response was triggered by adding 1.0 μg/mL of lipopolysaccharide from *E. coli* 0111:B4 (Sigma-Aldrich) to the pre-treated macrophages; the control cells were left without LPS treatment. After 24 h, the supernatants were collected and the concentrations of IL-1β and TNF-α were measured using a Human IL-1β and TNF-α ELISA Kit (Diaclone, Besançon, France), according to the manufacturer’s manual. Each experiment was run in triplicate.

#### 2.4.5. Detection of ROS

THP-1 cells in serum-free RPMI 1640 medium were seeded into 96-well plates (5 × 10^4^ cells/well) as described in the previous section. After 2 h of incubation, the cells were treated with test compounds **1**–**3**, **5**–**12**, and **13** (**a** + **b**) at a concentration of 5 μM. Thirty minutes later, pyocyanin at a final concentration of 100 μM was added to trigger oxidative stress in the cell culture. After another 30 min incubation at 37 °C, DCFH-DA (5 μg/mL) was added. Yet another 30 min incubation under the same conditions preceded spectrophotometric measurement of the fluorescent product DCF using a FLUOstar Omega Microplate Reader (BMG Labtech, Ortenberg, Germany) with λ (ex./em.) = 485/520 nm. Results were compared with those from the vehicle, which were established as 100%. The flavonoid quercetin at a concentration of 1 μM was used as the standard for comparison of the antioxidant properties of the test compounds.

#### 2.4.6. Statistical Evaluation

Statistical analyses were carried out using GraphPad Prism 6.01 software (San Diego, CA, USA). The data were graphed as means ± SEM. Comparisons between groups were made using a one-way ANOVA test followed by a Bonferroni’s multiple comparisons test.

## 3. Results

### 3.1. Isolation and Elucidation of Compounds

**Figure 1:**
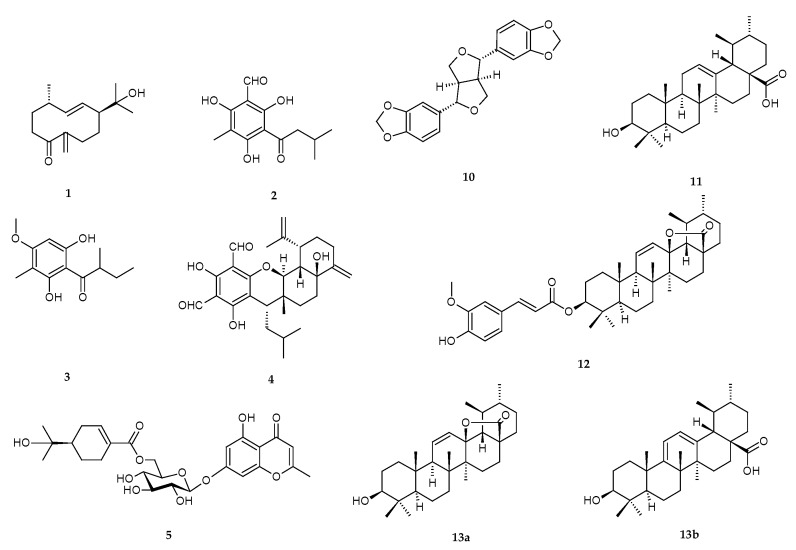

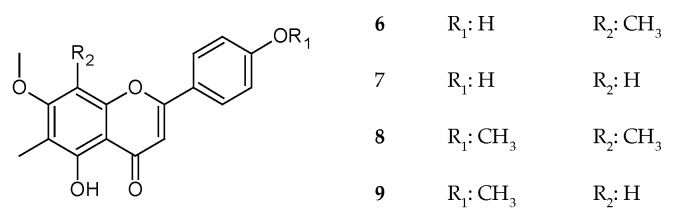
Structures of isolated compounds.

Chromatographic separation of the chloroform part of the ethanol extract of *E. globulus* leaves and twigs led to the isolation of 12 pure compounds and one mixture of two similar entities (Figure 1). Structural analyses based on comparison of the NMR, MS, IR and UV spectra with the literature resulted in the identification of litseagermacrane (**1**) [22], grandinol (**2**) [23], pulverulentone B (**3**) (also known as aspidinol D) [24], eucalyptal A (**4**) [25], cypellocarpin C (**5**) [26,27], sideroxylin (**6**) [28,29], 8-demethylsideroxylin (**7**) [28,30], eucalyptin (**8**) [31], 8-demethyleucalyptin (**9**) [31,32], sesamin (**10**) [32], ursolic acid (**11**) [32], tereticornate A (**12**) [33], and a mixture of the constitutional isomers ursolic acid lactone (**13**a) [32] and loxanic acid (**13**b) [34]. Litesagermacrane (**1**) is a monocyclic sesquiterpenoid, compounds **2**–**4** belong to prenylated phloroglucinols, **4**–**9** to C-methylated flavons, **11**–**13** to triterpenes. Compound **5** is a product of a biosynthetic combination of monoterpenic glycoside and a methylchromone. Sesamin (**10**) is a typical example of lignan.

### 3.2. Antiherpetic Activity

#### 3.2.1. Anti-HSV-1 Activity

Compounds **1**–**12** and **13** (**a** + **b**) were evaluated with respect to the antiviral potential of *Eucalyptus* extracts described in the literature. Of the isolated compounds, grandinol (**2**), sideroxylin (**6**), and tereticornate A (**12**) exerted significant inhibitory effects on the replication of HSV-1. Before performing the anti-HSV-1 assay, we assessed the cytotoxicity of each sample in Vero cells by the neutral red dye-uptake method. The CC_50_ values of active compounds **2**, **6**, and **12** along with acyclovir were found to be greater than 210 μg/mL (Table 1). The measured IC_50_ values showed that tereticornate A (**12**) (0.96 μg/mL) had the greatest anti-HSV-1 potential among the test compounds. Moreover, grandinol (**2**) and sideroxylin (**6**), with IC_50_ values of 1.23 and 1.44 μg/mL, respectively, were more active than acyclovir (1.92 μg/mL). Selectivity index CC_50_/IC_50_ is essential in identifying any possible toxic effect on the cell of a compound that could be confused with antiviral activity. Thus, a comparison of their selectivity index CC_50_/IC_50_ values: >218.8, >170.7, >145.8, and >109.4, respectively, shows that tereticornate A (**12**), grandinol (**2**), and sideroxylin (**6**) all possess greater anti-HSV-1 activity than acyclovir.

#### 3.2.2. Anti-HSV-2 Activity

Of the compounds evaluated for inhibitory effects on the replication of HSV-2, litseagermacrane (**1**) and cypellocarpin C (**5**) showed the greatest potential. The previously mentioned determination of cytotoxicity in Vero cells by the neutral red dye-uptake method preceded measurement of the anti-HSV-2 activity. The CC_50_ values of compounds **1** and **5** and acyclovir were found to be greater than 210 μg/mL (Table 2). The anti-HSV-2 activity was determined in infected Vero cells, by titer reduction assay using quantitative real-time reverse transcription PCR. Cypellocarpin C (**5**) and litseagermacrane (**1**) exerted greater anti-HSV-2 activity than acyclovir. The respective EC_50_ values were 0.73, 1.25, and 1.75 μg/mL, with corresponding selectivity index CC_50_/EC_50_ values of >287.7, >168 and >120 (Table 2).

### 3.3. Antibacterial and Antifungal Activity

The compounds isolated from the leaves and twigs of *E. globulus* were tested for the antimicrobial activity against the Gram-positive bacteria *S. aureus* and *B. cereus*, the Gram-negative bacteria *E. coli* and *P. aeruginosa* and the yeast *C. albicans*. Some compounds showed inhibitory activity of against both Gram-positive bacteria strains. Tetracycline and tioconazole were used as reference antibiotics. As presented in Table 3, compounds **2** and **3** showed moderate activities in the MIC range of 8–32 μg/mL against both Gram-positive strains. *B. cereus* was the most sensitive organism tested against the 8 of 13 compounds. No significant inhibition of growth was observed in the Gram-negative bacteria or the yeast even at the highest concentrations tested.

### 3.4. Cell Viability Assay of THP-1 Cells

**Figure 2:**
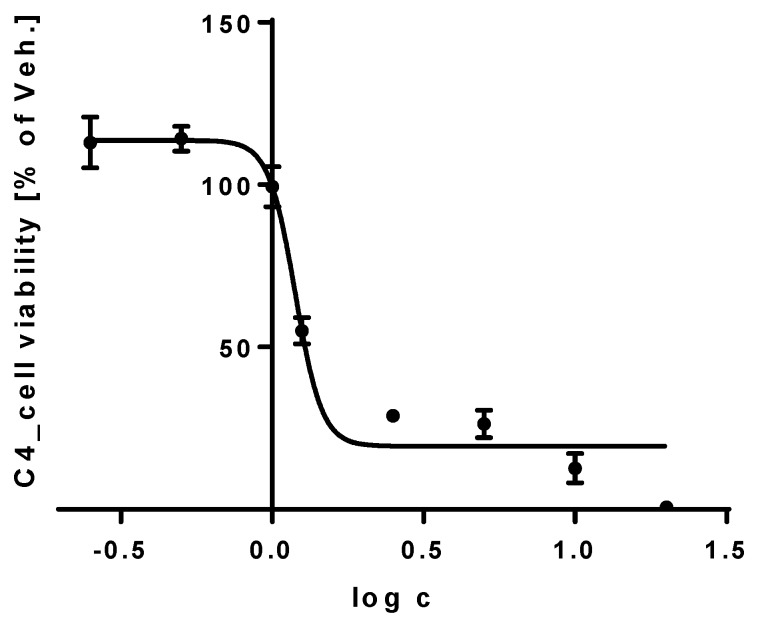
Effect of compound **4** on the viability of human leukemic monocytes THP-1. The cells were treated with compound **4** dissolved in DMSO at increasing concentrations 0.25–0.5–1–1.25–2.5–5–10–20 μM or with only the vehicle and their viability was measured after 24 h. Untreated control cells were used to verify the non-toxic effect of the vehicle. The viability of the cells treated with compound **4** was compared to that of the vehicle-treated cells. The results are expressed as the mean ± SE for three independent assays. The concentration of DMSO never exceeded 0.1%.

The effect of compounds **1**–**12** and **13** (**a** + **b**) on the viability of cells of THP-1 cell line was assessed using a WST-1 assay to detect metabolically active cells. The aim of the assay was to determine the concentration of each compound that would be safe, not influence the viability of cells and could be used for subsequent experiments. Cells were treated with the test compounds at concentrations increasing from 0.25 to 20 μM and the cell viability was measured spectrophotometrically after 24 h. All of the test compounds showed relatively safe profiles with IC_50_ value generally above 20 μM, except compound **4** (Figure 2) for which the IC_50_ for the THP-1 cell line was 1.2 μM (95% CI = 1.1–1.3 μM). Based on these results, compounds **1**–**3**, **5**–**12**, and **13** (**a** + **b**), which showed safe profiles, were chosen for subsequent measurements of the NF-κB/AP-1 and antioxidant activities. A non-toxic concentration of 5 μM was selected for these assays.

### 3.5. Anti-Inflammatory Activity

**Figure 3:**
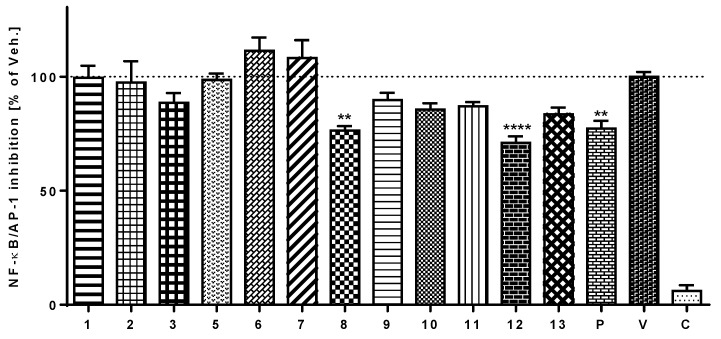
Effect of compounds **1**–**3**, **5**–**12**, and **13** (**a** + **b**) on NF-κB/AP-1 activity. THP-1-XBlue™-MD2-CD14 cells were pre-treated for 1 h with the test compounds (5 μM dissolved in DMSO), prednisone (P; 5 μM dissolved in DMSO), with only the vehicle (V). Subsequently, LPS (1 μg/mL) was added (to all but C, control cells) to trigger the activation of NF-κB/AP-1. After 24 h, the activity of NF-κB/AP-1 was evaluated based on the amount of secreted alkaline phosphatase measured spectrophotometrically. The results are expressed as the mean ± SE for six independent experiments measured in triplicate. ** indicates a significant difference (*p* < 0.01) in comparison with the vehicle-treated cells and **** indicates a significant difference (*p* < 0.0001) in comparison with the vehicle-treated cells.

Transcriptional factors NF-κB and AP-1 are involved in the response of the immune system to different stimuli, such as cytokines, cellular stress, or bacterial or viral infections. NF-κB controls many of the genes involved in inflammation and regulates the transcription of several pro-inflammatory proteins, e.g., TNF-α and IL-1β. The ability to attenuate the activation of NF-κB/AP-1 was determined using THP-1-XBlue™-MD2-CD14 cells. LPS-stimulation of the TLR4 receptors in this cell line induced signaling cascades that led to the activation of NF-κB and AP-1 and the subsequent production of the reporter protein SEAP, the activity of which was measured spectrophotometrically using a QUANTI-Blue™ assay. Several test compounds reduced the activation of NF-κB/AP-1 24 h after the addition of LPS (Figure 3). Compound **12** showed a statistically significant inhibitory potential, stronger than that of prednisone, a routinely used anti-inflammatory drug. Compound **8** also reduced the activity of NF-κB/AP-1 in a statistically significant manner (*p* < 0.05), comparable to prednisone.

**Figure 4:**
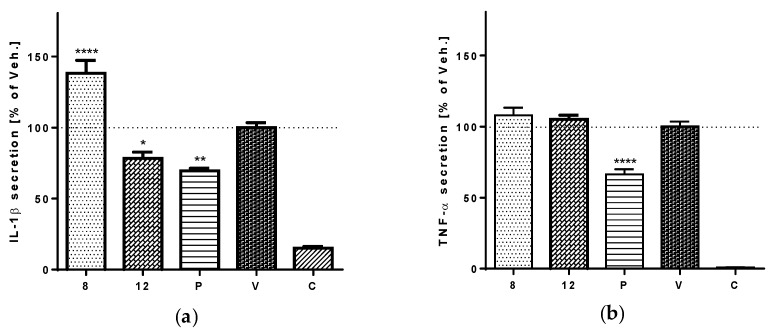
The effects of compounds **8** and **12** on the secretion of pro-inflammatory cytokines IL-1β (**a**) and TNF-α (**b**). PMA-differentiated macrophages were pre-treated with test compounds **8** and **12** at a concentration of 5 μM, with prednisone (P) 1 μM dissolved in DMSO, or with only the vehicle (V). LPS 1 μg/mL was added (to all but C, control cells) after 1 h of incubation. After 24 h, the supernatants were collected and the secretions of IL-1β and TNF-α in each culture medium were evaluated using ELISA. Results are expressed as the mean ± SE for three independent experiments. * indicates a significant difference (*p* < 0.05) in comparison with the vehicle-treated cells, ** indicates a significant difference (*p* < 0.01), and **** *p* < 0.0001.

Compounds **8** and **12** were the most active in the assay of NF-κB/AP-1 activity. Hereinafter, we aimed to show the effects of these two compounds on the LPS-stimulated secretion of pro-inflammatory cytokines IL-1β and TNF-α. This assay was carried out on PMA-differentiated THP-1 macrophages using ELISA. The results proved the anti-inflammatory potential of tereticornate A (**12**), which attenuates the LPS-stimulated secretion of IL-1β by inhibiting NF-κB. In contrast, eucalyptin (**8**) increased the secretion of the IL-1β (Figure 4a). Neither compound **8** nor **12** showed any influence on the secretion of TNF-α (Figure 4b).

### 3.6. Antioxidant and Pro-Oxidant Activity

**Figure 5:**
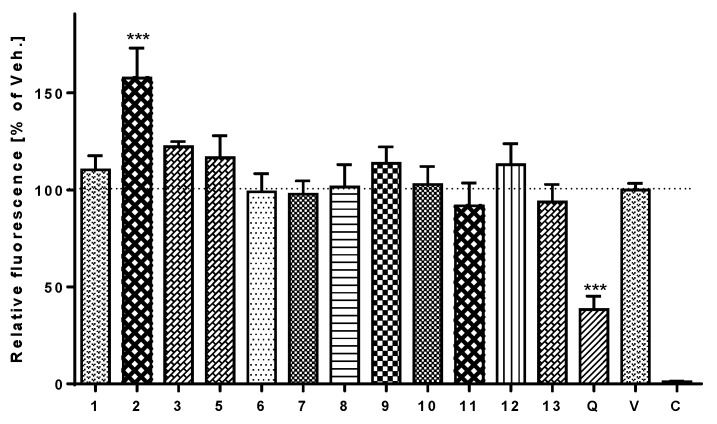
Effect of selected *E. globulus* compounds on the production of ROS. THP-1 monocytes were pre-treated with compounds **1**–**3**, **5**–**12**, and **13** (**a** + **b**) at concentrations of 5 μM, with 1 μM quercetin (Q) or with only the vehicle (V) for 30 min. Pyocyanin at a final concentration of 100 μM was then added (to all but C, control cells) to trigger the generation of ROS. After 30 min, DCFH-DA (5 μg/mL) dissolved in DMF was introduced into the cell medium. The intracellular fluorescence of dichlorofluorescein produced in this oxidative reaction was measured spectrophotometrically. The results in Figure 5 show the mean ± SE for three independent experiments. *** indicates a significant difference (*p* < 0.001) in comparison with the vehicle-treated cells.

The cell-permeant dye 2′,7′-dichlorodihydrofluorescein diacetate (DCFH-DA) was used to detect and determine any antioxidative or pro-oxidative effects of the test compounds. Pyocyanin, a toxin that is synthesized by G-negative *Pseudomonas aeruginosa* bacteria and possesses redox-active properties, was used to trigger oxidative stress in cells. Fluorescent 2′,7′-dichlorodihydrofluorescein (DCF), a product of direct oxidation by reactive oxygen species (ROS), was measured spectrophotometrically. The flavonoid quercetin at a concentration of 1 μM was used as a standard antioxidant for comparison. Test compounds **1**, **3**, **5**–**12**, and **13** (**a** + **b**) at a concentration of 5 μM showed no significant antioxidant potential, but compound **2** significantly increased the production of ROS in THP-1 cells (Figure 5).

## 4. Discussion

As visible from an overview of isolated compounds, we were able to obtain several groups of metabolites with majority of *C*-methylated flavones, typical for *Eucalyptus* (**6**–**9**) and triterpenes (**11**–**13**). The enumeration is then refilled with phloroglucinols **2**–**4**, lignan (**10**), and a combination of monoterpenic glycoside and a methylchromone (**5**).

The isolation of compounds **3** and **12** from the *E. globulus* is reported here for the first time. Compounds **1**, **4**, and **5** had previously been described only in the fruits of *E. globulus* [25,35]. We propose that litseagermacrane (**1**) may be a biogenetic precursor of the cadinane-type intermediate involved in the biosynthesis of eucalyptal A (**4**). In our study, compound **4** showed a cytotoxic effect against THP-1 cells and similar activity with IC_50_ 1.7 μM has been reported against HL-60 (promyelocytic leukemia) cells [25]. Eucalyptal A (**4**) belongs to the large chemical group of phloroglucinol-terpene adducts typical for *Eucalyptus* species, that comprises different types of compounds, e.g., euglobals or macrocarpals. Euglobals exhibited numerous of pharmacological activities—such as anti-tumor promoting, antimicrobial, antileishmanial activity—and they also inhibited activation of Epstein–Barr virus, another member of the Herpesviridae family. Some macrocarpals has also shown antiviral activity against HIV-reverse transcriptase [16].

The testing of biological activity was limited by the limited amounts of isolated compounds. It will be essential to yield greater amounts for the detailed investigation that is required for the complex evaluation of the antiviral potential. An isolation of natural compounds from the plant material is a demanding and time-consuming process based on consequent steps of repeated extraction, liquid–liquid separation, normal-phase liquid chromatography and preparative high-performance chromatography using reverse-phase column. Because we revealed the compounds with antiviral and anti-inflammatory activity, the antiviral research of non-volatile compounds of *E. globulus* will continue after further isolation process.

No compound isolated from *E. globulus* and possessing antiherpetic activity has yet been described in the literature. Of the test compounds, tereticornate A (**12**) showed the strongest inhibitory potential against HSV-1. Moreover, its antiviral effect was approximately twice as strong as that of acyclovir, the standard medicament in clinical use. The chemical group of pentacyclic triterpenes has already shown the antiviral properties. For example, betulin, lupeol, and betulinic acid, as well as triterpene extract obtained from birch bark, have exhibited antiviral activity against different strains of HSV-1, including KOS [36]. In addition to compound **12**, grandinol (**2**) also showed promising activity against HSV-1. It had already shown antiviral activity against the activation of Epstein–Barr virus, another member of the Herpesviridae family [5,12]. Despite the considerable structural similarities of the *C*-methylated flavonoids tested—compounds **6**–**9**—only sideroxylin (**6**) showed any activity against HSV-1. Some compounds from this chemical family have exerted antiviral activity against the H1N1 influenza virus [37]. The most active test compound against HSV-2 was cypellocarpin C (**5**), with activity more than twice as potent as that of acyclovir; but litseagermacrane (**1**) also showed a potent antiherpetic effect. This compound had already been found to inhibit the replication of HIV-1 in a green fluorescent protein-based reporter cell line [22]. These results show that tereticornate A (**12**) and cypellocarpin C (**5**) are good candidates for the further antiherpetic research and maybe potentially become antiherpetic drugs.

Acylphloroglucinols **2** and **3** showed significant antibacterial activities against both of the Gram-positive strains measured. Only these two compounds were active against *S. aureus*, but *B. cereus* was sensitive to several of the test compounds. In addition to compounds **2** and **3**, compound **4** and the mixture **13** (**a** + **b**) also showed antibacterial activities against *B. cereus*. The antibacterial activity of grandinol (**2**) against both Gram-positive strains is well known from the literature [38]. The antibacterial activity of grandinol (**2**) could also correlate with the generation of ROS in THP-1 monocytes that was noticed in the antioxidant assay. Antimicrobial agents often generate ROS that then rupture the bacterial plasmatic membrane and allow the agent to penetrate within bacteria [39]. Free radicals attack essential cellular components of microbes, including lipids, proteins and DNA [40]. Pulverulentone B (**3**), isolated from *Hypericum chinense* as aspidinol D, has shown antibacterial activity against the *S. aureus*, but has shown even stronger action against the NorA efflux overexpressing multidrug-resistant *S. aureus* strain than against the standard *S. aureus* ATCC 25923 [24]. Acylphloroglucinols are phenolic compounds with different levels of methylation biosynthesized via acetate pathway [16]. Grandinol (2) and pulverulenton B (3) are known as inhibitors of cress germination [41].

Knowing, that related compounds isolated from *Piliostigma thonningii* had antimicrobial activity against *S. aureus* NCTC 6571 [42], we expected positive assay results for all of the *C*-methylated flavonoids we isolated. However, our *C*-methylated flavonoids showed little or no effects on the bacterial strains tested. Compounds **8** and **9** showed no positive effect, although both compounds had been active against the strains *S. aureus* IFO12732 and *B. cereus* IFO3001 [4]. We suppose that these different results may have been caused by the different bacterial strains than in our study or different methodology.

Tereticornate A (**12**) has shown a statistically significant ability to inhibit the activation of NF-κB/AP-1, that is greater than that of prednisone, a routinely used anti-inflammatory drug. Tereticornate A (**12**) also attenuates the LPS-stimulated secretion of IL-1β. Compound **12** has been found to actively inhibit the infectivity of HSV-1 in Vero cells and has no significant cytotoxic effects. NF-κB is a crucial regulator of host transcription. It can be activated by HSV-1 infection and can initiate the inflammatory process in the host [43]. Our results show that tereticornate A (**12**) significantly inhibits the activation of NF-κB and we suggest that the anti-inflammatory effect of this compound may be mediated by suppression of the HSV-1-induced NF-κB. Triterpenic compounds could be responsible for the anti-inflammatory properties of *E. globulus*, but further analyses would be needed to support this thesis. The *C*-methylated flavonoids represent a rare category of flavonoids that have at least one methyl group attached directly to a carbon atom of an aromatic ring. This group comprises relatively lipophilic compounds. Eucalyptin (**8**) showed promising activity by reducing the activation of NF-κB/AP-1 in a statistically significant manner, comparable to that of prednisone. Furthermore, the results for the *C*-methylated flavonoids have uncovered the interesting fact, that eucalyptin (**8**) and 8-demethyleucalyptin (**9**) reduce the activation of NF-κB/AP-1, whereas sideroxylin (**6**) and 8-demethylsideroxylin (**7**) enhance it. Based on comparison of the molecular structures of the test flavonoids, we supposed that the crucial factor for the activity is the substituent attached to carbon C-4′ of the B-ring of the flavone. In compounds **8** and **9**, this position is occupied by a methoxyl substituent. The inhibitory effect on the activation of NF-κB/AP-1 disappears when the methoxyl group is replaced by a hydroxyl substituent. Comparing the activities and structures of compounds **8** and **9**, we can deduce that the methyl group attached to the carbon C-8 of eucalyptin (**8**) contributes to its inhibitory effect on the activation of NF-κB/AP-1. This is probably due to the greater lipophilicity of the molecule which easies its penetration through a cell membrane. The secretion of TNF-α was not affected by neither **8** nor **12**, which showed the greatest inhibition effect on NF-κB/AP-1, but the level of IL-1β was modified. Its expression was reduced by **12**, which is in the agreement with observed lower NF-κB/AP-1 activity. On the other hand, **8** significantly increased the production of IL-1β. It can be caused by prolonged transcription of *IL-1β* gene without effect on *TNF-α* gene, which was described for some methylated flavonoids [44].

## 5. Conclusions

Fourteen compounds of different structural types were obtained from an ethanolic extract of *E. globulus* by chromatographic separation. The therapeutic use of eucalyptus oil against viral infections and local inflammations inspired us to analyze the isolated compounds for their antiviral activity against the replication of HSV-1 and HSV-2. Their antimicrobial effects on several Gram-positive and Gram-negative bacterial strains and one fungus strain were also determined, along with their anti-inflammatory activities in cell-based assays. Several of the test compounds (**1**, **2**, **5**, **6**, and **12**) showed antiviral activity with potentials greater than acyclovir, along with moderate antibacterial effects and anti-inflammatory activity. The combined results show the promise of *Eucalyptus* compounds as leads for the therapy of some viral infections.

## Figures and Tables

**Table 1:** Anti-HSV-1 activity and cytotoxicity of active compounds **2**, **6**, and **12**.

Compound	CC_50_ (μg/mL)	IC_50_ (μg/mL)	Selectivity Index CC_50_/IC_50_
**2**	>210	1.23 ± 0.13	>170.7
**6**	>210	1.44 ± 0.14	>145.8
**12**	>210	0.96 ± 0.12	>218.8
Acyclovir	>210	1.92 ± 0.23	>109.4

Values presented are means ± SD of three to five independent experiments performed in duplicates, CC_50_: 50% cytotoxic concentration, IC_50_: 50% inhibitory concentration.

**Table 2:** Anti-HSV-2 activity and cytotoxicity of active compounds **1** and **5**.

Compound	CC_50_ (μg/mL)	EC_50_ (μg/mL)	Selectivity Index CC_50_/EC_50_
**1**	>210	1.25 ± 0.20	>168.0
**5**	>210	0.73 ± 0.11	>287.7
Acyclovir	>210	1.75 ± 0.33	>120.0

Values presented are means ± SD of three independent experiments performed in duplicate, CC_50_: 50% cytotoxic concentration, EC_50_: 50% effective concentration.

**Table 3:** MIC values (μg/mL) of compounds isolated from *E. globulus*.

Compound	*S. aureus* ATCC 29213	*B. cereus* ATCC 11778	*E. coli* ATCC 25922	*P. aeruginosa* ATCC 27853	*C. albicans* ATCC 10231
**1**	>64	**64**	>64	>64	>64
**2**	**16**	**16**	>64	>64	>64
**3**	**32**	**8**	>32	>32	>32
**4**	>128	**32**	>128	>128	>128
**5**	>128	>128	>128	>128	>128
**6**	>64	**64**	>64	>64	>64
**7**	>128	**128**	>128	>128	>128
**8**	>64	>64	>64	>64	>64
**9**	>64	>64	>64	>64	>64
**10**	>32	>32	>32	>32	>32
**11**	**128**	**32**	>128	>128	>128
**12**	>32	>32	>32	>32	>32
**13** (**a** + **b**)	**128**	**16**	**128**	**128**	**128**
**Antibiotic**	0.125 ^a^	0.03 ^a^	1 ^a^	16 ^a^	4 ^b^

^a^ tetracycline, ^b^ tioconazole.

## Supplementary Materials

The following are available online at http://www.mdpi.com/1999-4915/10/7/360/s1, Figure S1: COSY and NOESY scheme of litseagermacrane (**1**), Figure S2: HMBC and COSY scheme of grandinol (**2**), Figure S3: HMBC, COSY and NOESY scheme of pulverulentone B (**3**), Figure S4: COSY and NOESY scheme of eucalyptal A (**4**), Figure S5: HMBC and COSY scheme of cypellocarpin C (**5**), Figure S6: HMBC, COSY and NOESY scheme of sideroxylin (**6**), Figure S7: HMBC, COSY and NOESY scheme of 8-demethylsideroxylin (**7**), Figure S8: HMBC, COSY and NOESY scheme of eucalyptin (**8**), Figure S9: HMBC, COSY and NOESY scheme of 8-demethyleucalyptin (**9**), Figure S10: COSY and NOESY scheme of sesamin (**10**), Figure S11: COSY and NOESY scheme of ursolic acid (**11**), Figure S12: COSY and NOESY scheme of tereticornate A (**12**), Figure S13: COSY and NOESY scheme of ursolic acid lactone (**13a**), Figure S14: COSY and NOESY scheme of loxanic acid (**13b**), Table S1: ^1^H and ^13^C chemical shifts (δ in ppm) and HMBC correlations of litseagermacrane (**1**), Table S2: ^1^H and ^13^C chemical shifts (δ in ppm) and HMBC correlations of grandinol (**2**), Table S3: ^1^H and ^13^C chemical shifts (δ in ppm) and HMBC correlations of pulverulentone B (**3**), Table S4: ^1^H and ^13^C chemical shifts (δ in ppm) and HMBC correlations of eucalyptal A (**4**), Table S5: ^1^H and ^13^C chemical shifts (δ in ppm) and HMBC correlations of cypellocarpin C (**5**), Table S6: ^1^H and ^13^C chemical shifts (δ in ppm) and HMBC correlations of sideroxylin (**6**), Table S7: ^1^H and ^13^C chemical shifts (δ in ppm) and HMBC correlations of 8-demethylsideroxylin (**7**), Table S8: ^1^H and ^13^C chemical shifts (δ in ppm) and HMBC correlations of eucalyptin (**8**), Table S9: ^1^H and ^13^C chemical shifts (δ in ppm) and HMBC correlations of 8-demethyleucalyptin (**9**), Table S10: ^1^H and ^13^C chemical shifts (δ in ppm) and HMBC correlations of sesamin (**10**), Table S11: ^1^H and ^13^C chemical shifts (δ in ppm) and HMBC correlations of ursolic acid (**11**), Table S12: ^1^H and ^13^C chemical shifts (δ in ppm) and HMBC correlations of tereticornate A (**12**), Table S13: ^1^H and ^13^C chemical shifts (δ in ppm) and HMBC correlations of ursolic acid lactone (**13a**), Table S14: ^1^H and ^13^C chemical shifts (δ in ppm) and HMBC correlations of loxanic acid (**13b**).
