# Supplementary material for: Anti-Infectivity against Herpes Simplex Virus and Selected Microbes and Anti-Inflammatory Activities of Compounds Isolated from Eucalyptus globulus Labill
Source: Viruses. 2018 Jul 6;10(7):360. doi: 10.3390/v10070360 (PMC6070903; doi:10.3390/v10070360)
Supplement: Supplementary file 1 [file viruses-10-00360-s001.pdf]

Supplementary Materials:

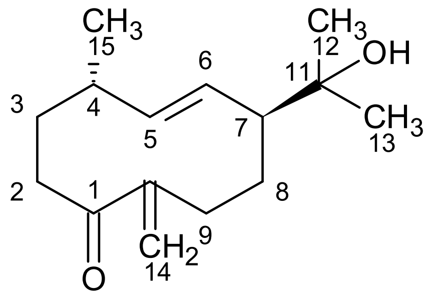

Litseagermacrane

Table S1. ^{1}H and ^{13}C chemical shifts (δ in ppm) and HMBC correlations of litseagermacrane (**1**).

Position	δC (ppm), type of atom	δH (ppm), mult. (*J* in Hz)	HMBC correlations
1	206.0, C		
2	37.4, CH_2	2.30, m	1, 3, 4
		2.83, m	1, 3, 4
3	36.8, CH_2	1.80, m	2, 4
		2.01, m	2, 4
4	40.6, CH	2.08, m	2, 5, 6
5	142.7, CH	5.00, dd (15.27, 9.33)	4, 6, 7, 15
6	130.1, CH	5.05, dd (15.40, 9.46)	4, 5, 7
7	56.9, CH	1.92, m	5, 6, 8, 9, 11, 12, 13
8	32.7, CH_2	1.30, m	6, 7, 9, 10, 11
		1.35, m	6, 7, 9, 10, 11
9	28.7, CH_2	2.25, m	7, 10, 14
		2.55, t (13.00)	7, 8, 10, 14
10	155.0, C		
11	71.6, C		
12	26.7, CH_3	1.09, s	7, 11, 13
13	26.7, CH_3	1.13, s	7, 11, 12
14	119.7, CH_2	5.50, s	1, 9, 10
		5.64, s	1, 9, 10
15	20.6, CH_3	0.97, d (6.00)	3, 4, 5

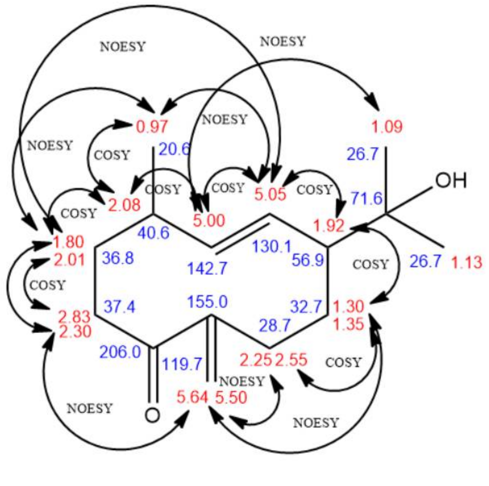

Figure S1. COSY and NOESY scheme of litseagermacrane (**1**).

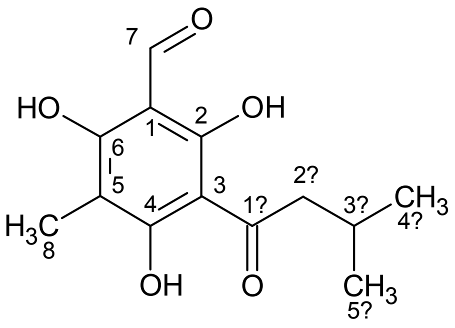

Grandinol

Table S2. ^{1}H and ^{13}C chemical shifts (δ in ppm) and HMBC correlations of grandinol (**2**).

Position	δC (ppm), type of atom	δH (ppm), mult. (*J* in Hz)	HMBC correlations
1	102.8, C		
2	167.7, C		
3	103.9, C		
4	171.4, C		
5	100.5, C		
6	160.9, C		
7	191.2, CHO	10.12, s	2
8	6.16, CH_3	2.05, s	4, 5, 6
1′	206.6, C		
2′	52.7, CH_2	3.01, 2H, d (6.55)	1′, 3′, 4′, 5′
3′	24.9, CH	2.27, m	2′, 4′, 5′
4′	22.6, CH_3	0.99, d (6.55)	2′, 3′, 5′
5′	22.6, CH_3	0.99, d (6.55)	2′, 3′, 4′
2-OH		14.34, s	1, 2, 3
4-OH		15.46, s	3, 4, 5

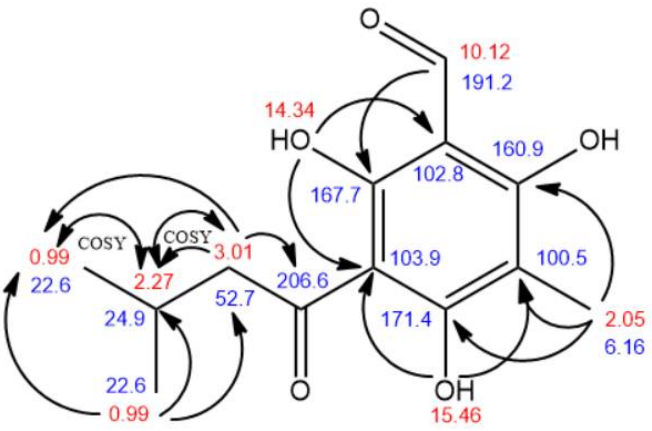

Figure S2. HMBC and COSY scheme of grandinol (**2**)

.

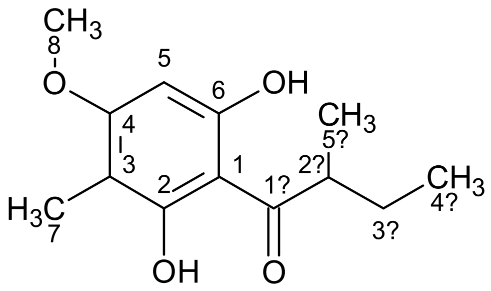

Pulverulentone B

Table S3. ^{1}H and ^{13}C chemical shifts (δ in ppm) and HMBC correlations of pulverulentone B (**3**).

**Position**	**δC (ppm), type of atom**	**δH (ppm), mult. (*J* in Hz)**	**HMBC correlations**
1	105.5, C		
2	165.0, C		
3	102.8, C		
4	161.0, C		
5	89.7, CH	5.93, s	1, 3, 4, 6
6	159.3, C		
7	7.04, CH_3	2.05, s	2, 3, 4
8	55.2, OCH_3	3.85, s	4
1′	210.2, C		
2′	46.1, CH	3.63, m	1′, 5′
3′	27.1, CH_2	1.38, m	1′, 2′, 4′, 5′
		1.78, m	1′, 2′, 4′, 5′
4′	12.3, CH_3	0.91, dd (7.67)	2′, 3′
5′	16.9, CH_3	1.15, d (6.73)	1′, 2′, 3′
2-OH		14.34	1, 2, 3
6-OH		12.79	

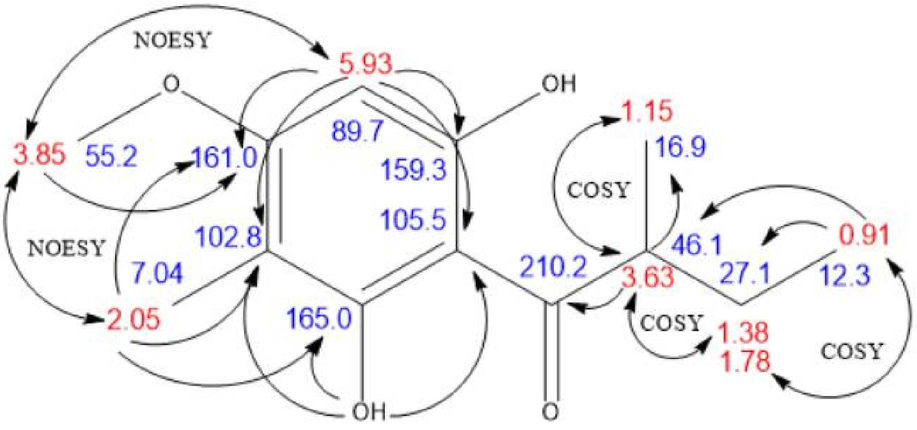

Figure S3. HMBC, COSY, and NOESY scheme of pulverulentone B (**3**).

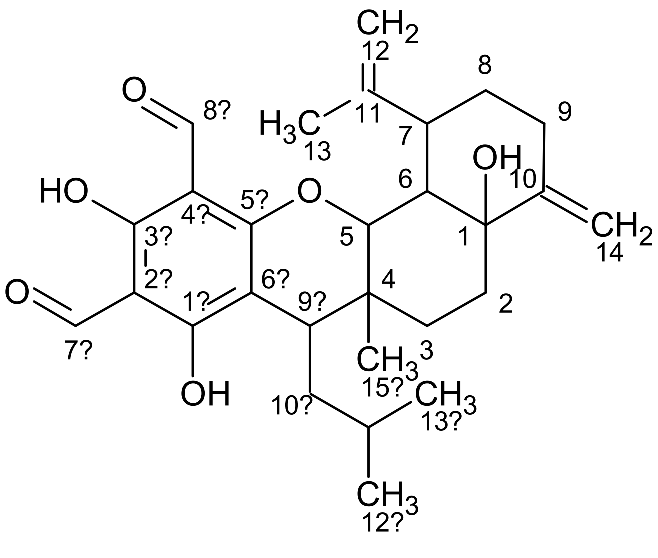

Eucalyptal A

Table S4. ^{1}H and ^{13}C chemical shifts (δ in ppm) and HMBC correlations of eucalyptal A (**4**).

Position	δC (ppm), type of atom	δH (ppm), mult. (*J* in Hz)	HMBC correlations
1	73.2, C		
2	30.3, CH_2	2.21, m	1, 4, 6
		2.46, m	1, 4, 6
3	29.7, CH_2	1.40, m	1, 4, 5, 15
		1.88, m	1, 2, 4, 5, 15
4	35.7, C		
5	75.4, CH	4.07, d (11.64)	6, 15
6	44.6, CH	2.60, dd (2.75, 11.59)	2, 5, 7, 8, 10
7	39.5, CH	3.12, br d (11.97)	5, 6, 11
8	23.4, CH_2	1.79, m	7, 9, 10
		1.96, m	9, 11
9	32.1, CH_2	2.41, m	1, 7, 8, 14
		2.66, m	1, 7, 8, 14
10	146.5, C		
11	149.4, C		
12	107.8, CH_2	4.72, s	7, 13
		4.84, s	7, 13
13	22.3, CH_3	1.87, s	7, 11, 12
14	111.1, CH_2	5.01, s	1, 9
		5.11, s	1, 9
15	19.6, CH_3	0.98, s	3, 4, 5, 9′
1′	168.5, C		
2′	104.0, C		
3′	167.1, C		
4′	103.9, C		
5′	162.6, C		
6′	107.5, C		
7′	191.2, CHO	10.15, s	1′, 2′, 8′
8′	194.9, CHO	9.89, s	3′, 4′, 7′
9′	37.6, CH	2.49, dd (8.61, 1.80)	5, 5′, 6′, 10′, 11′
10′	42.8, CH_2	1.09, m	6′, 9′

		1.32, m	9′
11′	28.4, CH	1.64, m	-
12′	21.6, CH_3	1.02, d (6.42)	10′, 11′, 13′
13′	22.7, CH_3	0.88, d (6.42)	10′, 11′, 12′
1′-OH		13.29, s	1′, 2′, 6′
3′-OH		13.75, s	2′, 3′, 4′

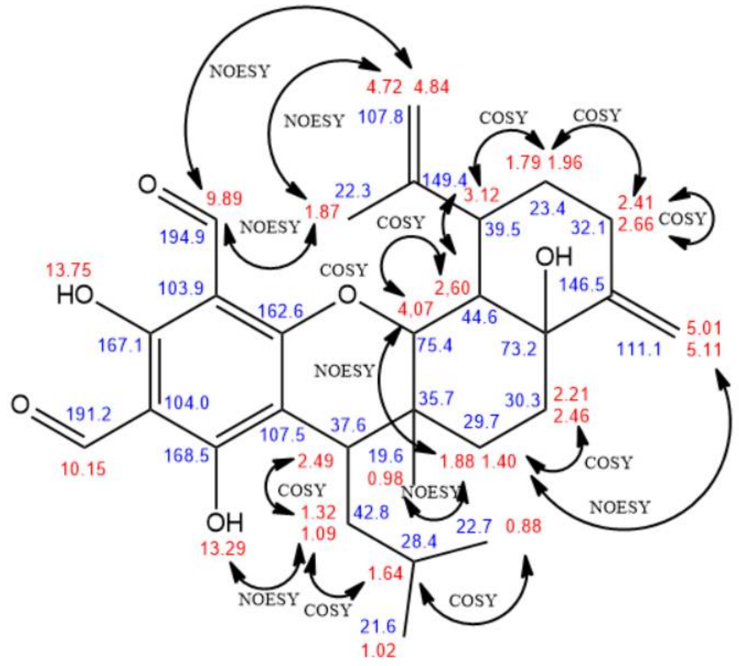

Figure S4. COSY and NOESY scheme of eucalyptal A (**4**).

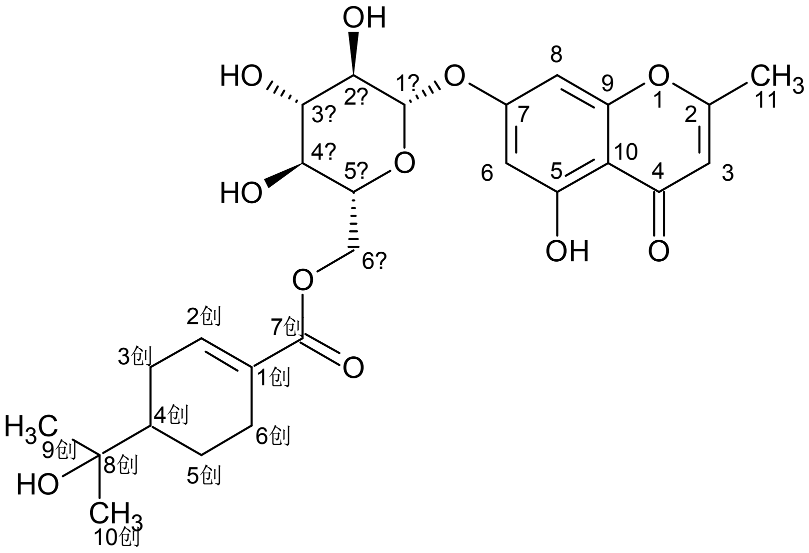

Cypellocarpin C

Table S5. ^{1}H and ^{13}C chemical shifts (δ in ppm) and HMBC correlations of cypellocarpin C (**5**).

Position	δC (ppm), type of atom	δH (ppm), mult. (*J* in Hz)	HMBC correlations
2	168.5, C		
3	108.5, CH	6.26, s	2, 10
4	182.1, C		
5	162.3, C		
6	99.3, CH	6.43, d (2.00)	5, 7, 8, 10
7	162.4, C		
8	94.5, CH	6.63, d (2.00)	6, 7, 9, 10
9	157.7, C		
10	104.7, C		
11	20.5, CH_3	2.39, s	2, 3
1′	99.3, CH	5.12, dd (3.20, 7.50)	7
2′	73.3, CH	3.27, m	
3′	76.4, CH	3.30, m	
4′	70.4, CH	3.16, m	
5′	74.1, CH	3.78, m	
6′	63.9, CH_2	3.97, dd (7.88, 12.17)	
		4.42, m	
1′′	134.8, C		
2′′	140.4, CH	6.91, m	1′′, 4′′, 7′′
3′′	27.4, CH_2	1.92, m	
		2.20, m	
4′′	43.8, CH	1.35, m	
5′′	23.2, CH_2	1.04, m	
		1.86, m	
6′′	25.2, CH_2	2.01, m	
		2.36, m	
7′′	165.8, C		
8′′	70.3, C		
9′′	27.1, CH_3	1.04, s	4′′, 8′′, 10′′
10′′	27.1, CH_3	1.04, s	4′′, 8′′, 9′′
5-OH		12.84, s	5, 6, 10

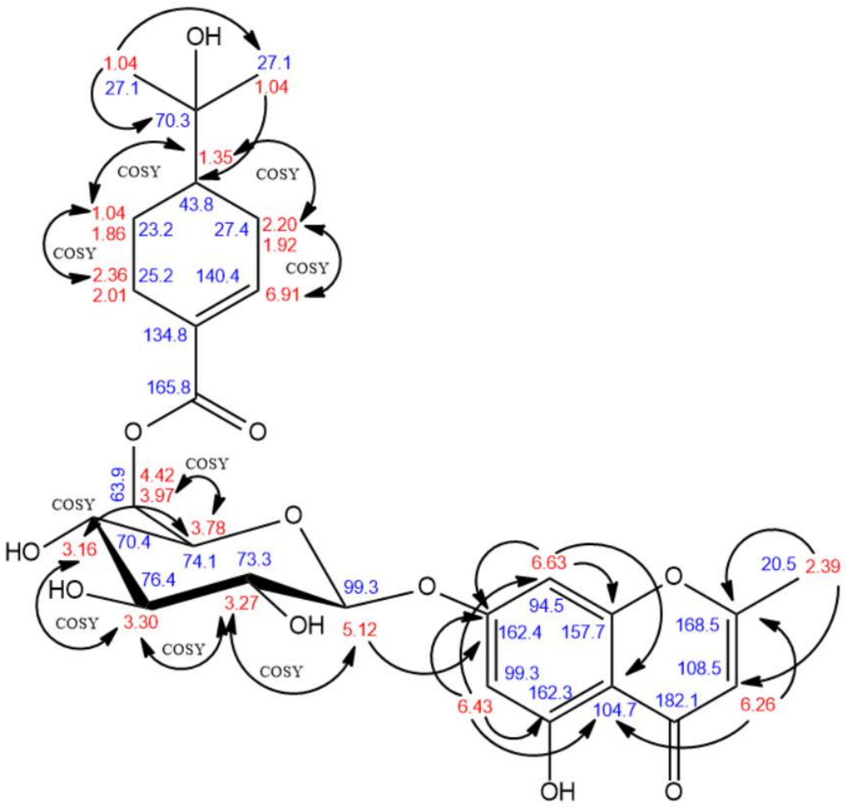

Figure S5. HMBC and COSY scheme of cypellocarpin C (**5**).

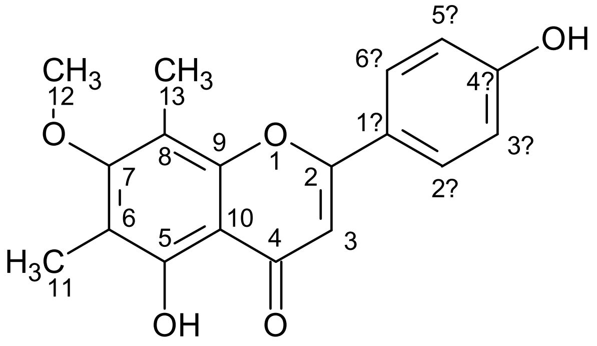

Sideroxylin

Table S6. ^{1}H and ^{13}C chemical shifts (δ in ppm) and HMBC correlations of sideroxylin (**6**).

Position	δC (ppm), type of atom	δH (ppm), mult. (*J* in Hz)	HMBC correlations
2	164.2, C		
3	103.1, CH	6.89, s	1′, 2, 4, 10
4	182.9, C		
5	156.5, C		
6	113.2, C		
7	162.4, C		
8	109.1, C		
9	152.4, C		
10	107.3, C		
11	8.5, CH_3	2.11, s	5, 6, 7
12	60.8, OCH_3	3.76, s	7
13	8.6, CH_3	2.35, s	7, 8, 9
1′	121.6, C		
2′	128.9, CH	7.97, d (8.80)	2, 4′, 6′
3′	116.4, CH	6.96, d (8.80)	1′, 4′, 5′
4′	162.2, C		
5′	116.4, CH	6.96, d (8.80)	1′, 3′, 4′
6′	128.9, CH	7.97, d (8.80)	2, 2′, 4′
5-OH		13.08, s	
4′-OH		10.39, br s	

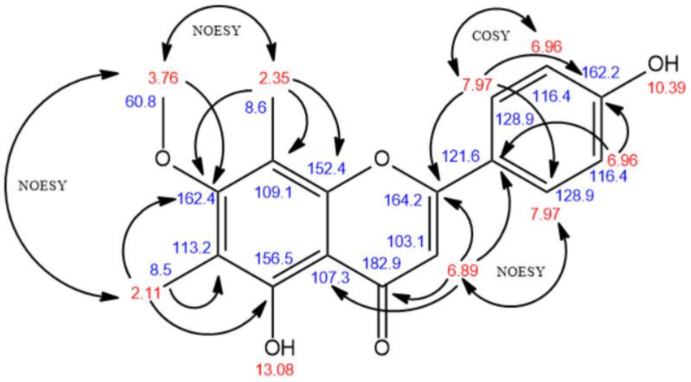

Figure S6. HMBC, COSY, and NOESY scheme of sideroxylin (**6**).

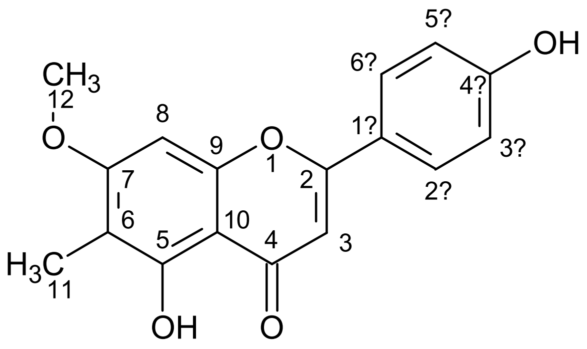

8-Demethylsideroxylin

Table S7. ^{1}H and ^{13}C chemical shifts (δ in ppm) and HMBC correlations of 8-demethylsideroxylin (**7**).

Position	δC (ppm), type of atom	δH (ppm), mult. (*J* in Hz)	HMBC correlations
2	164.3, C		
3	103.1, CH	6.85, s	1′, 2, 4, 10
4	182.5, C		
5	158.7, C		
6	107.8, C		
7	163.3, C		
8	89.2, CH	6.87, s	6, 7, 9, 10
9	155.6, C		
10	104.4, C		
11	7.9, CH_3	2.01, s	5, 6, 7
12	56.7, OCH_3	3.93, s	7
1′	121.1, C		
2′	128.8, CH	7.97, d (8.78)	2, 4′, 6′
3′	115.6, CH	6.94, d (8.78)	1, 4′, 5′
4′	161.1, C		
5′	115.6, CH	6.94, d (8.78)	1′, 3′, 4′
6′	128.8, CH	7.97, d (8.78)	2, 2′, 4′
5-OH		13.11, s	
4′-OH		10.35, br s	

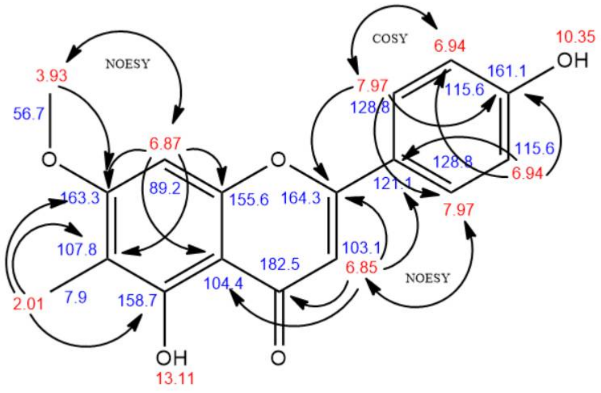

Figure S7. HMBC, COSY, and NOESY scheme of 8-demethylsideroxylin (**7**).

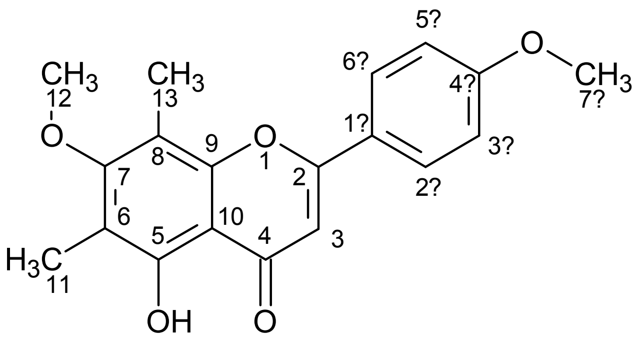

Eucalyptin

Table S8. ^{1}H and ^{13}C chemical shifts (δ in ppm) and HMBC correlations of eucalyptin (**8**).

Position	δC (ppm), type of atom	δH (ppm), mult. (*J* in Hz)	HMBC correlations
2	163.9, C		
3	103.7, CH	6.64, s	1′, 2, 4, 10
4	183.0, C		
5	156.9, C		
6	114.0, C		
7	162.4, C		
8	108.4. C		
9	152.5, C		
10	107.1, C		
11	8.1, CH_3	2.21, s	5, 6, 7
12	61.2, OCH_3	3.81, s	7
13	9.3, CH_3	2.41, s	7, 8, 9
1′	123.6, C		
2′	127.7, CH	7.89, d (8.74)	4′, 6′
3′	114.0, CH	7.05, d (8.74)	1′, 4′, 5′
4′	162.7, C		
5′	114.0, CH	7.05, d (8.74)	1′, 3′, 4′
6′	127.7, CH	7.89, d (8.74)	2′, 4
7′	55.6, OCH_3	3.91, s	4′
5-OH		12.88, br s	

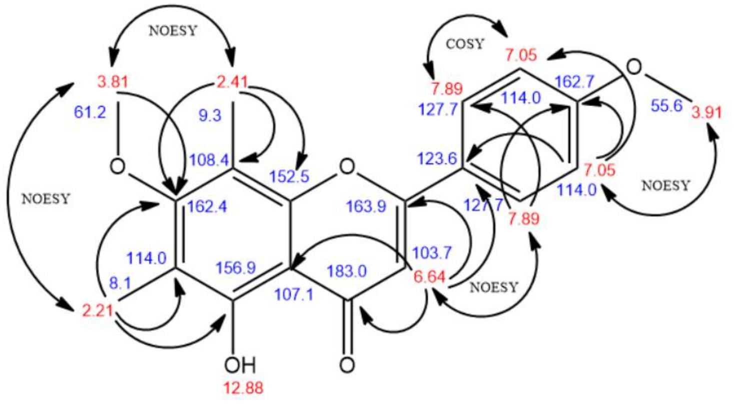

Figure S8. HMBC, COSY, and NOESY scheme of eucalyptin (**8**).

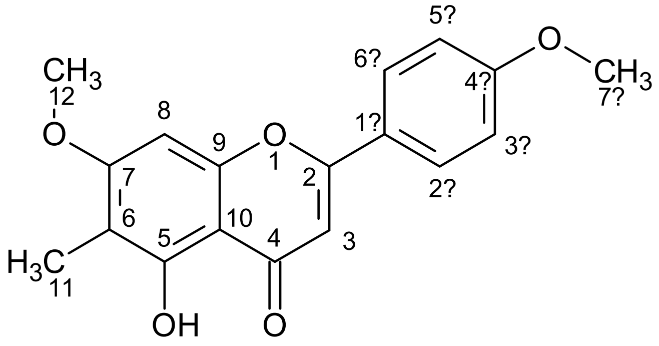

8-Demethyleucalyptin

Table S9. ^{1}H and ^{13}C chemical shifts (δ in ppm) and HMBC correlations of 8-demethyleucalyptin (**9**).

Position	δC (ppm), type of atom	δH (ppm), mult. (*J* in Hz)	HMBC correlations
2	163.3, C		
3	104.4, CH	6.61, s	1′, 2, 4, 10
4	182.4, C		
5	157.9, C		
6	108.9, C		
7	162.4, C		
8	89.2, CH	6.51, s	6, 7, 9, 10
9	155.9, C		
10	105.1, C		
11	7.2, CH_3	2.13, s	5, 6, 7
12	55.7, OCH_3	3.94, s	7
1′	123.8, C		
2′	127.8, CH	7.86, d (8.92)	4′, 6′
3′	114.3, CH	7.03, d (8.92)	1′, 4′, 5′
4′	162.1, C		
5′	114.3, CH	7.03, d (8.92)	1′, 3′, 4′
6′	127.8, CH	7.86, d (8.92)	2′, 4′
7′	55.3, OCH_3	3.91, s	4′

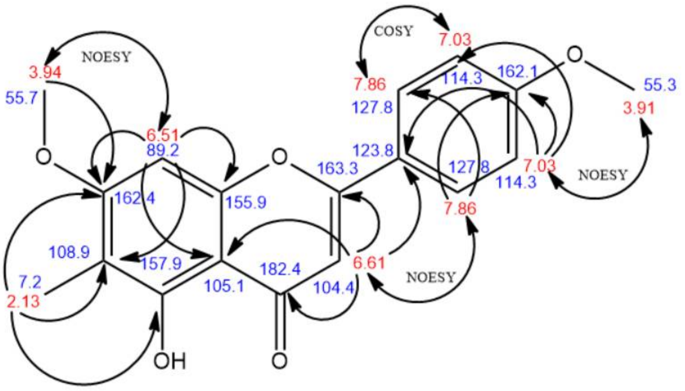

Figure S9. HMBC, COSY, and NOESY scheme of 8-demethyleucalyptin (**9**).

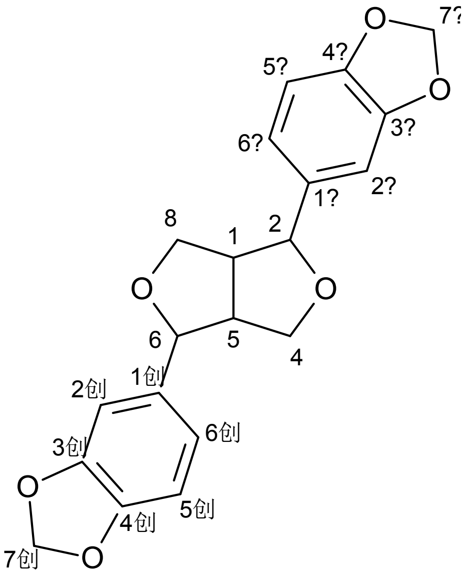

Sesamin

Table S10. ^{1}H and ^{13}C chemical shifts (δ in ppm) and HMBC correlations of sesamin (**10**).

Position	δC (ppm), type of atom	δH (ppm), mult. (*J* in Hz)	HMBC correlations
1	54.2, CH	3.06, m	
2	85.6, CH	4.73, d (4.39)	1, 8, 1′, 2′, 6′
4	71.5, CH_2	3.89, m	6
		4.24, m	5, 6
5	54.2, CH	3.06, m	
6	85.6, CH	4.73, d (4.39)	4, 5, 1′′, 2′′, 6′′
8	71.5, CH_2	3.89, m	2
		4.24, m	1, 2
1′	134.2, C		
2′	106.0, CH	6.86, d (1.23)	2, 4′, 6′
3′	147.6, C		
4′	146.3, C		
5′	107.9, CH	6.78, obscured	1′, 3′
6′	119.1, CH	6.80, obscured	1′, 2′, 4′
7′	100.6, CH_2	5.96, 2H, s	3′, 4′
1′′	134.2, C		
2′′	106.0, CH	6.86, d (1.23)	6, 4′′, 6′′
3′′	147.6, C		
4′′	146.3, C		
5′′	107.9, CH	6.78, obscured	1′′, 3′′
6′′	119.1, CH	6.80, obscured	1′′, 2′′, 4′′
7′′	100.6, CH_2	5.96, 2H, s	3′′, 4′′

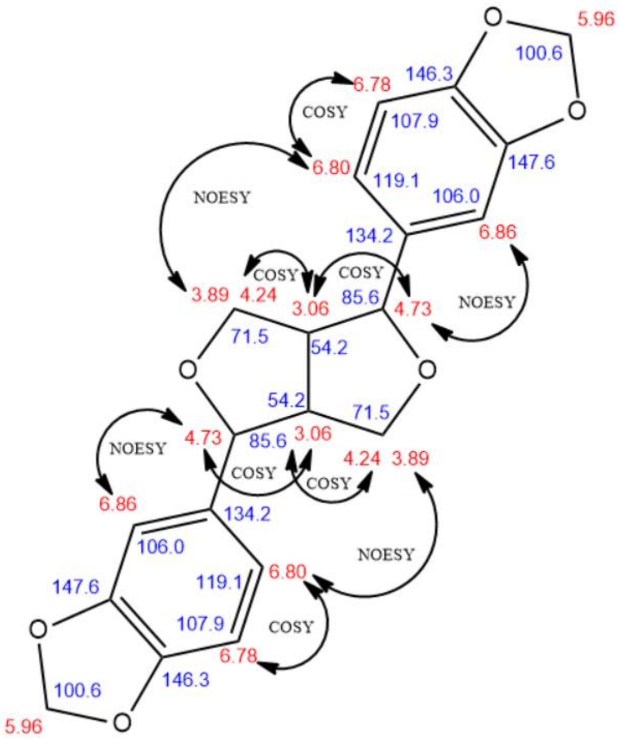

Figure S10. COSY and NOESY scheme of sesamin (**10**).

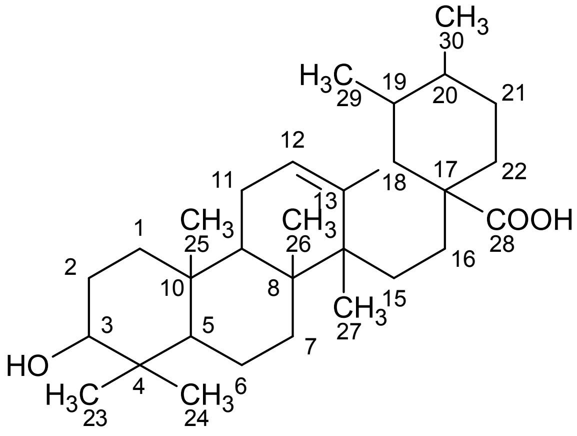

Ursolic acid

Table S11. ^{1}H and ^{13}C chemical shifts (δ in ppm) and HMBC correlations of ursolic acid (**11**).

**Position**	**δC (ppm), type of atom**	**δH (ppm), mult. (*J* in Hz)**	**HMBC correlations**
1	36.4, CH_2	1.29, m	
		1.54, m	2, 3, 5, 10
2	27.4, CH_2	1.28, m	
		1.45, m	
3	77.2, CH	3.00, m	4, 23, 24
4	38.8, C		
5	55.2, CH	0.68, m	
6	18.5, CH_2	1.30, m	
		1.40, m	
7	32.9, CH_2	1.23, m	
		1.38, m	
8	39.7, C		
9	47.5, CH	1.45, m	1, 8, 9, 11, 14
10	36.4, C		
11	23.4, CH_2	1.84, 2H, m	
12	124.7, CH	5.13, t (3.02)	9, 11, 14, 18
13	138.6, C		
14	42.1, C		
15	28.4, CH_2	0.74, m	
		1.79, m	
16	23.8, CH_2	1.50, m	
		1.92, m	
17	47.6, C		
18	52.7, CH	2.11, d (11.53)	12, 13, 14, 17, 20, 28, 29
19	38.8, CH	1.30, m	
20	39.0, CH	0.92, m	21
21	30.5, CH_2	1.26, m	
		1.45, m	
22	39.9, CH_2	1.90, m	
		2.10, m	
23	16.4, CH_3	0.68, s	3, 4, 5, 24
24	28.6, CH_3	0.89, s	3, 4, 5, 23
25	21.5, CH_3	0.87, s	1, 9
26	16.7, CH_3	0.75, s	7, 8, 9, 14

27	23.6, CH_3	1.04, s	8, 14, 15
28	178.8, C		
29	17.0, C	0.81, obscured	18, 19
30	28.2, C	0.92, obscured	19, 21

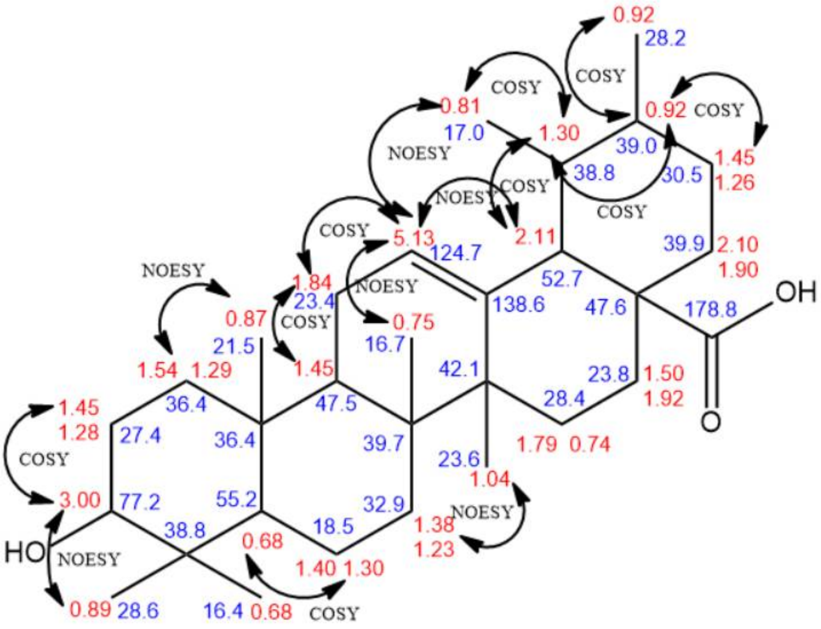

Figure S11. COSY and NOESY scheme of ursolic acid (**11**).

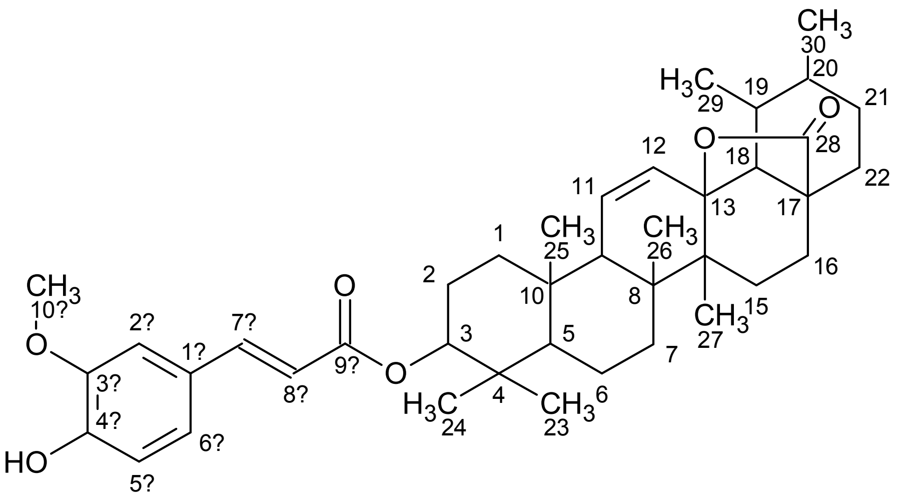

Tereticornate A

Table S12. ^{1}H and ^{13}C chemical shifts (δ in ppm) and HMBC correlations of tereticornate A (**12**).

Position	δC (ppm), type of atom	δH (ppm), mult. (*J* in Hz)	HMBC correlations
1	31.0, CH_2	1.13, m	
		1.82, m	
2	25.5, CH_2	1.24, m	
		1.74, m	
3	80.3, CH	4.66, dd (5.79, 10.50)	9′
4	37.6, C		
5	54.7, CH	0.90, m	10
6	17.5, CH_2	1.64, 2H, m	
7	30.5, CH_2	1.61, m	
		1.80, m	
8	41.5, C		
9	52.9, CH	2.01, s	10, 12, 14
10	36.9, C		
11	128.8, CH	5.55, dd (2.25, 10.30)	8, 9
12	133.0, CH	5.96, d (10.30)	13, 14
13	89.5, C		
14	41.5, C		
15	25.1, CH_2	1.23, m	
		1.74, m	
16	22.9, CH_2	1.44, m	
		2.16, m	28
17	45.0, C		
18	60.4, CH	1.65, m	13, 16, 17, 19
19	40.2, CH	0.92, m	
20	38.1, CH	1.81, m	
21	30.4, CH_2	1.27, m	
		1.72, m	
22	31.3, CH_2	1.23, m	
		1.57, m	
23	27.6, CH_3	0.92, s	3, 4, 5, 24
24	16.3, CH_3	0.96, s	3, 4, 5, 23
25	17.8, CH_3	0.98, s	1, 9, 10

26	18.2, CH_3	1.08, s	7, 9, 14
27	16.2, CH_3	1.18, s	8, 13, 15
28	180.0, C		
29	19.1, CH_3	0.95, d (6.13)	18, 19, 20
30	17.6, CH_3	1.02, d (6.13)	19, 20, 21
1′	126.6, C		
2′	108.9, CH	7.05, s	4′, 6′
3′	146.7, C		
4′	147.6, C		
5′	114.5, CH	6.94, d (8.33)	1′, 3′
6′	122.6, CH	7.10, d (8.33)	2′, 4′
7′	144.3, CH	7.61, d (16.00)	2′, 6′, 9′
8′	115.9, CH	6.29, d (16.00)	1′, 9′
9′	166.7, C		
10′	55.9, OCH_3	3.95, s	3′

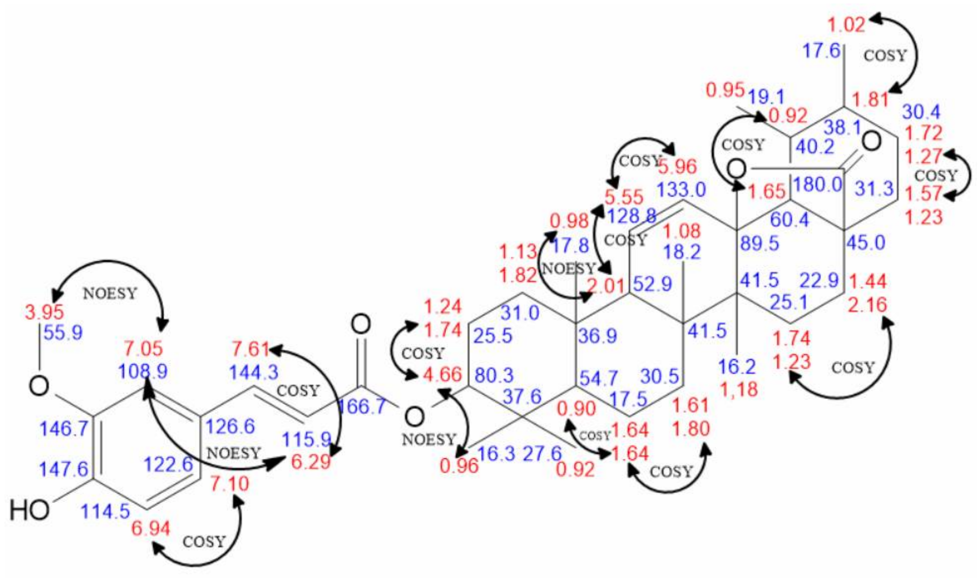

Figure S12. COSY and NOESY scheme of tereticornate A (**12**).

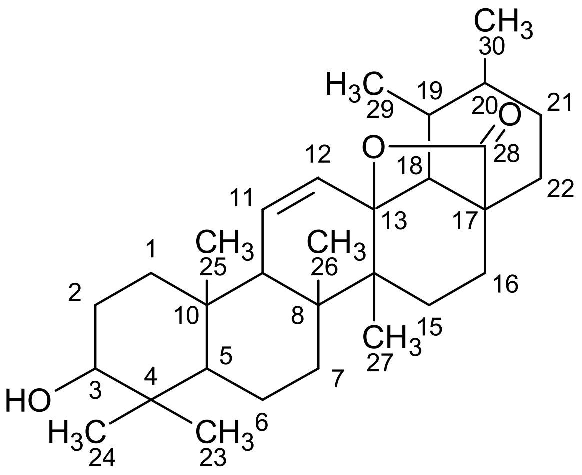

Ursolic acid lactone

Table S13. ^{1}H and ^{13}C chemical shifts (δ in ppm) and HMBC correlations of ursolic acid lactone (**13a**).

Position	δC (ppm), type of atom	δH (ppm), mult. (*J* in Hz)	HMBC correlations
1	37.3, CH_2	1.36, m	
		2.03, m	
2	27.0, CH_2	1.63, m	
		1.69, m	
3	78.6, CH	3.23, m	
4	38.5, C		
5	54.3, CH	0.75, m	
6	17.6, CH_2	1.48, m	
		1.59, m	
7	30.9, CH_2	1.63, m	
		1.82, m	
8	41.3, C		
9	52.4, CH	1.96, m	
10	36.2, C		
11	133.3, CH	5.98, m	8, 10, 13
12	128.6, CH	5.54, m	
13	89.7, C		
14	41.6, C		
15	25.0, CH_2	1.68, m	
		1.79, m	
16	22.4, CH_2	1.40, m	
		2.15, m	15, 17, 28
17	43.7, C		
18	60.2, CH	1.64, m	12, 13, 17, 19, 29
19	38.3, CH	1.32, m	17, 18
20	38.5, CH	1.78, m	
21	30.4, CH_2	1.29, m	
		1.54, m	
22	36.9, CH_2	1.34, m	
		1.80, m	
23	14.7, CH_3	0.78, s	3, 4, 5, 24
24	27.2, CH_3	0.99, s	3, 4, 5, 23

25	16.8, CH_3	0.90, s	5, 9, 10
26	19.0, CH_3	1.06, s	7, 9, 14
27	15.9, CH_3	1.16, s	8, 13, 15
28	179.7, C		
29	17.8, CH_3	1.00, obscured	18, 19, 20
30	18.4, CH_3	0.93, obscured	

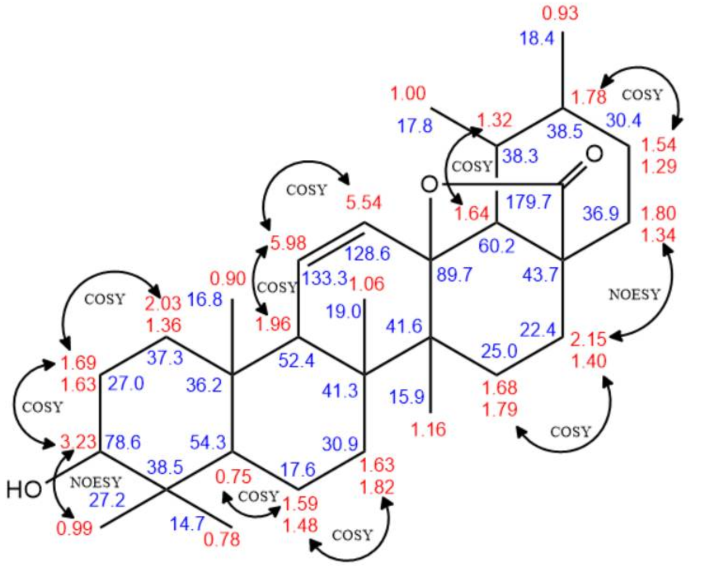

Figure S13. COSY and NOESY scheme of ursolic acid lactone (**13a**).

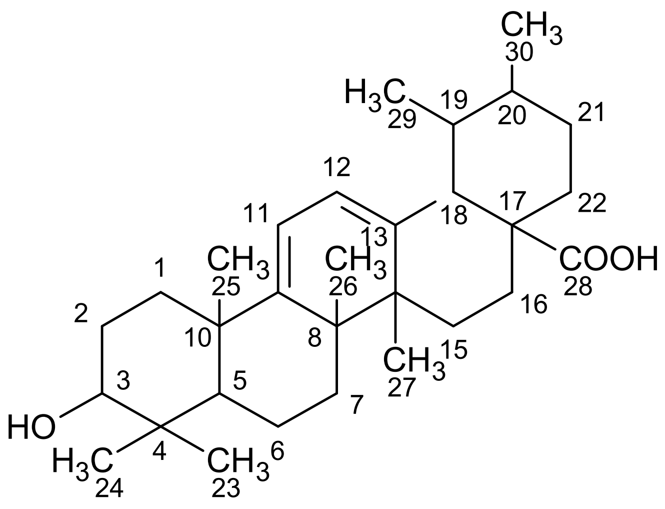

3β-hydroxyursa-9(11),12-dien-28-oic acid (loxanic acid)

Table S14. ^{1}H and ^{13}C chemical shifts (δ in ppm) and HMBC correlations of loxanic acid (**13b**).

**Position**	**δC (ppm), type of atom**	**δH (ppm), mult. (*J* in Hz)**	**HMBC correlations**
1	36.4, CH_2	obscured	
2	27.0, CH_2	1.63, m	
		1.69, m	
3	78.6, CH	3.23, m	
4	38.5, C		
5	50.9, CH	0.84, m	
6	17.6, CH_2	1.48, m	
		1.59, m	
7	32.1, CH_2	1.63, m	
		1.82, m	
8	42.9, C		
9	154.7, C		
10	38.6, C		
11	115.1, CH	5.59, obscured	8, 9, 10, 13
12	123.1, CH	5.52, d (2.63)	9, 11, 14, 18
13	138.6, C		
14	40.6, C		
15	26.9, CH_2	1.69, m	
		2.03, m	
16	24.6, CH_2	1.69, m	
		1.74, m	
17	47.2, C		
18	50.7, CH	2.32, m	12, 13, 14, 16, 17, 19
19	38.5, CH	1.32, m	
20	38.5, CH	1.78, m	
21	30.4, CH_2	1.29, m	
		1.54, m	
22	36.9, CH_2	1.36, m	28
		2.03, m	28
23	15.1, CH_3	0.79, s	3, 4, 5, 24
24	27.2, CH_3	1.02, s	3, 4, 5, 23

25	15.9, CH_3	1.17, s	1, 5, 9, 10
26	21.3, CH_3	0.98, s	7, 8, 9, 14
27	17.9, CH_3	0.92, s	8, 13, 14, 15
28	182.2, C		
29	15.1, CH_3	0.80, obscured	18, 19, 20
30	18.4, CH_3	0.94, obscured	

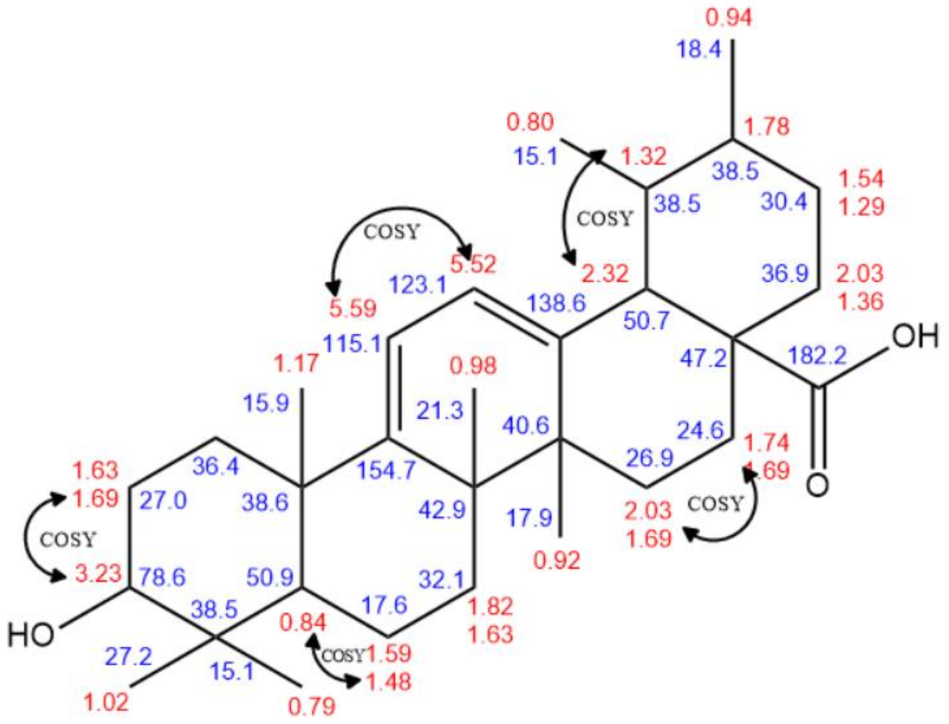

Figure S14. COSY scheme of loxanic acid (**13b**).
